# A maximum pseudo-likelihood approach for estimating species trees under the coalescent model

**DOI:** 10.1186/1471-2148-10-302

**Published:** 2010-10-11

**Authors:** Liang Liu, Lili Yu, Scott V Edwards

**Affiliations:** 1 Department of Agriculture and Natural Resources, Delaware State University, Dover, DE 19901, USA; 2 Department of Biostastistics, Georgia Southern University, Statesboro, GA 30460, USA; 3 Department of Organismic and Evolutionary Biology, Harvard University, Cambridge, MA 02138, USA

## Abstract

**Background:**

Several phylogenetic approaches have been developed to estimate species trees from collections of gene trees. However, maximum likelihood approaches for estimating species trees under the coalescent model are limited. Although the likelihood of a species tree under the multispecies coalescent model has already been derived by Rannala and Yang, it can be shown that the maximum likelihood estimate (MLE) of the species tree (topology, branch lengths, and population sizes) from gene trees under this formula does not exist. In this paper, we develop a pseudo-likelihood function of the species tree to obtain maximum pseudo-likelihood estimates (MPE) of species trees, with branch lengths of the species tree in coalescent units.

**Results:**

We show that the MPE of the species tree is statistically consistent as the number *M *of genes goes to infinity. In addition, the probability that the MPE of the species tree matches the true species tree converges to 1 at rate *O*(*M *^-1^). The simulation results confirm that the maximum pseudo-likelihood approach is statistically consistent even when the species tree is in the anomaly zone. We applied our method, Maximum Pseudo-likelihood for Estimating Species Trees (MP-EST) to a mammal dataset. The four major clades found in the MP-EST tree are consistent with those in the Bayesian concatenation tree. The bootstrap supports for the species tree estimated by the MP-EST method are more reasonable than the posterior probability supports given by the Bayesian concatenation method in reflecting the level of uncertainty in gene trees and controversies over the relationship of four major groups of placental mammals.

**Conclusions:**

MP-EST can consistently estimate the topology and branch lengths (in coalescent units) of the species tree. Although the pseudo-likelihood is derived from coalescent theory, and assumes no gene flow or horizontal gene transfer (HGT), the MP-EST method is robust to a small amount of HGT in the dataset. In addition, increasing the number of genes does not increase the computational time substantially. The MP-EST method is fast for analyzing datasets that involve a large number of genes but a moderate number of species.

## Background

Empirical studies on the evolutionary history of sequences from multiple loci show strong evidence of incongruent gene trees across loci [1-3]. Such incongruence challenges the appropriateness of traditional methods for estimating phylogenies, such as supermatrix approaches, which are based on the assumption that all loci have the same gene tree [4,5]. Several methods have been developed to accommodate the variability among gene trees when estimating species trees [6-11]. Consensus tree methods are commonly used to summarize a collection of gene trees [12-15] and can be easily adapted for the purpose of estimating species trees. Other approaches for combining multiple gene trees include supertree [16-18] and reconciliation [19-22] methods, which may capture important biological details through parameters without modelling it explicitly [23,24]. We here focus on species tree reconstruction methods developed in the context of the multispecies coalescent model [10,25-27], which assumes that gene trees are generated from the coalescent processes occurring in each branch of a species tree.

The relationship between gene coalescence times and species divergence times under the multispecies coalescent model has motivated several approaches for estimating species trees based on summary statistics of gene coalescence times [6,28-32]. Although the likelihood function of the species tree under the multispecies coalescent model has already been derived by Rannala and Yang [25], research on the maximum likelihood (ML) estimation of species trees under the coalescent remains limited [7,33,34]. Under a simplified multispecies coalescent model in which population sizes are assumed constant across populations, Liu et al. [32] have shown that the Maximum Tree, a species tree with the largest possible branch lengths under the constraint that gene coalescence times across loci always predate species divergence times, is the maximum likelihood estimate (MLE) of the species tree.

Previous studies have demonstrated, empirically and theoretically, strong evidence that species trees and gene trees should be considered as distinct quantities that describe two closely related evolutionary processes. A species tree represents the evolutionary pathway of species-usually the relevant goal in phylogenetic studies [35] - while a gene tree represents the evolutionary history of a single gene. This insight on the relationship between gene trees and the species tree provides a biological foundation for building probabilistic models to estimate species trees from gene trees. In the multispecies coalescence model [25,27,36], a gene tree is viewed as a coalescence process of genealogical lineages along branches in the species tree. Specifically, Rannala and Yang [25] showed that the probability distribution of the topology of gene tree *k *and the (*m_ik_-n_ik_*) coalescent time intervals $t_{ik}^{n_{ik}+1},...,t_{ik}^{m_{ik}}$ for population *i *reduced from *m_ik _*to *n_ik _*sampled lineages along a branch of length *τ_i _*in the species tree is

(1) $$\exp\left\{-\frac{n_{ik}\left(n_{ik}-1\right)}{\theta_{i}}\left(\tau_{i}-\sum_{j=n_{ik}+1}^{m_{ik}}t_{ik}^{j}\right)\right\}\times \prod_{j=n_{ik}+1}^{m_{ik}}\left\{\frac{2}{\theta_{i}}\exp\left(-\frac{j\left(j-1\right)}{\theta_{i}}t_{ik}^{j}\right)\right\},$$

where *θ*_*i *_= 4*α*_*i*_*μ *and *τ_i _*= *μβ_i_*; *μ *is the number of mutations per site per generation, *α_i _*is the effective population size of population *i*, and *β_i _*is the number of generations that population *i *extended over history. The second term (the product) in (1) is the probability of (*m_ik_-n_ik_*) coalescent time intervals (times between coalescent events) and the first term is the probability that *n_ik _*genealogical lineages do not coalesce in population *i*. For a collection of gene trees **G **that are independent of each other given the species tree, we multiply (1) across gene trees to find the likelihood for population *i *[25],

(2) $$\prod_{k=1}^{M}\left\{\exp\left(-\frac{n_{ik}\left(n_{ik}-1\right)}{\theta_{i}}\left(\tau_{i}-\sum_{j=n_{ik}+1}^{m_{ik}}t_{ik}^{j}\right)\right)\prod_{j=n_{ik}+1}^{m_{ik}}\left\{\frac{2}{\theta_{i}}\exp\left(-\frac{j(j-1)}{\theta_{i}}t_{ik}^{j}\right)\right\}\right\}$$

in which *M *is the number of genes. The probability density function *f*(**G**|*S*) of gene trees **G **given the species tree *S *is the product of (2) across all populations (branches of the species tree). The likelihood for population *i *in (2) can be simplified as

(3) $${\left(2/\theta_{i}\right)}^{a_{i}}e^{-b_{i}/\theta_{i}}$$

where

(4) $$b_{i}=\sum_{k=1}^{M}\left\{n_{ik}\left(n_{ik}-1\right)\left(\tau_{i}-\sum_{j=n_{ik}+1}^{m_{ik}}t_{ik}^{j}\right)+\sum_{j=n_{ik}+1}^{m_{ik}}\left\{j\left(j-1\right)t_{ik}^{j}\right\}\right\}\;and\;a_{i}=\sum_{k=1}^{M}\left(m_{ik}-n_{ik}\right).$$

Here coalescence time intervals $t_{ik}^{j}$ s are fixed because gene trees **G **are given. In addition, *b_i _*is bounded because the branch length *τ*_*i *_in the species tree is restricted due to the assumption that gene coalescence times always predate species divergence times.

**Figure 1:**
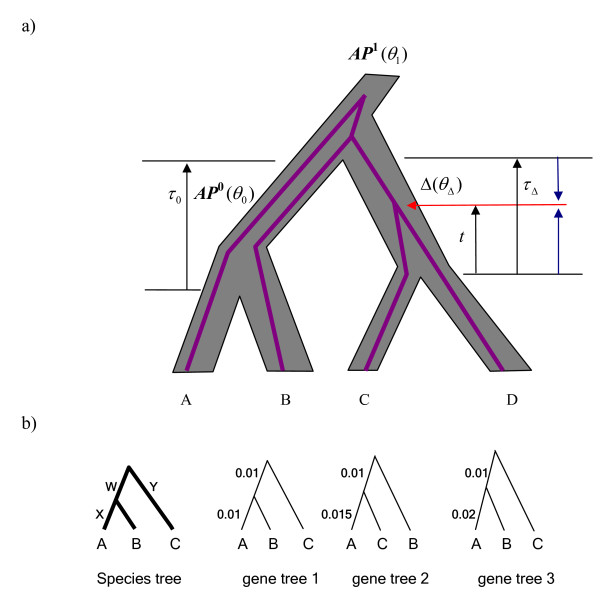
**The MLE of the species tree under the likelihood function f(G|S)**. a) The species tree (shaded) has three ancestral populations. The ancestral population *AP*^1 ^at the root of the tree is the common ancestral population of species A, B, C, and D. Two internal branches with length *τ*_0 _and *τ*_Δ _in the species tree represent the ancestral populations *AP*^0 ^and Δ. Population *AP*^0 ^is the common ancestral population of species A and B, while Δ is the common ancestral population of species C and D. Each population has a population size *θ *and branch length *τ*. The purple lines represent a gene tree, the evolutionary history of sequences sampled from species A, B, C, and D. The red arrow indicates the coalescence of the two sequences sampled from species C and D. The corresponding coalescence time interval is *t*, which decreases to 0 as branch length *τ*_Δ _decreases to 0 in the direction indicated by the two blue arrows. b) The likelihood of the species tree given three gene trees. The species tree and three gene trees are ultrametric trees. The number on each branch represents the branch length. In the species tree, *W *is the length of the internal branch and *Y *= *X*+*W*, *θ*_1 _is the population size for the root population, and *θ*_2 _is the population size for the ancestral population of species A and B.

We next show that the MLE of the species tree (topology, branch lengths, and population sizes) under the likelihood function *f*(**G**|*S*) we just described does not exist. Given a set of gene trees **G**, the MLE of the species tree is obtained by maximizing the likelihood function *f*(**G**|*S*) with respect to the topology, branch lengths, and population sizes of the species tree. Consider an ancestral population Δ in which two lineages in a gene tree coalesce (Figure 1a). Let *C *denote this coalescence event (indicated by the red arrow in Figure 1a). If multiple coalescence events involve in population Δ, *C *represents the first coalescence event occurring in population Δ Let the branch length *τ*_Δ _of population Δ go to zero while keeping the coalescence *C *within the population (the branch shrinks as indicated by the blue arrows in Figure 1a), then we have *a*Δ = 1 and *b*Δ → 0. Let *θ*_Δ_= *b*, which implies *θ*_Δ _→ 0. The likelihood of population Δ in (3) then becomes (2/*θ*_Δ_)*e*^-1 ^which goes to infinity as *θ*_Δ _0. Moreover, if we fix the population sizes for the populations other than Δ in the species tree, it follows from (3) that when the population size *θi *is fixed, the likelihood of population *i *is always greater than a positive number, because *b_i _*in (3) is bounded. Thus the likelihoods of the populations other than Δ are always greater than a positive number. Since the likelihood of the species tree *f*(**G**|*S*) is the product of the likelihoods for single populations, it goes to infinity as the likelihood of population Δ goes to infinity, while at the same time the likelihoods for other populations are always greater than a positive number. Note that for any gene trees we can always find an ancestral population Δ in a species tree such that the likelihood of the species tree goes to infinity as *θ*_Δ _→ 0, *b*_Δ _→ 0, and *θ*_Δ _= *b*Δ. The likelihood of the species tree is maximized (if we define $\infty =\frac{1}{0}$

As an example, we calculate the likelihood scores of species trees for three fixed gene trees ((A:0.01, B:0.01):0.01, C:0.02), ((A:0.015, C:0.015):0.01, B:0.025), and ((A:0.02, B:0.02):0.01, C:0.03) (Figure 1b). The species tree is ((A:*X*, B:*X*):*W*, C:*Y*) where *X *is the divergence time of species A and B and *Y *is the height of the species tree. Note that *Y *= *X*+*W *because the species tree is ultrametric (Figure 1b). Branch lengths in the species tree and gene trees are in mutation units. Let *θ*_1 _be the population size of the root population and *θ*_1 _be the population size on the branch with length *W *in the species tree. Due to the constraint that gene coalescence times should be strictly larger than species divergence times, we have *X *< 0.01 and *Y *< 0.015. Let 0.01 <*Y *< 0.015. The likelihood of the species tree is

$\begin{array}{l} \overset{\text{gene tree }1}{\overset{︷}{\left(\frac{2}{\theta_{2}}e^{-\frac{2(0.01-X)}{\theta_{2}}}\times \frac{2}{\theta_{1}}e^{-\frac{2(0.02-Y)}{\theta_{1}}}\right)}}\times \overset{\text{gene tree 2}}{\overset{︷}{\left(e^{-\frac{2W}{\theta_{2}}}\times \frac{2}{\theta_{1}}e^{-\frac{6(0.015-Y)}{\theta_{1}}}\times \frac{2}{\theta_{1}}e^{-\frac{2(0.025-0.015)}{\theta_{1}}}\right)}}\times \overset{\text{gene tree 3}}{\overset{︷}{\left(e^{-\frac{2W}{\theta_{2}}}\times \frac{2}{\theta_{1}}e^{-\frac{6(0.02-Y)}{\theta_{1}}}\times \frac{2}{\theta_{1}}e^{-\frac{2(0.03-0.02)}{\theta_{1}}}\right)}}\\ =\frac{2}{\theta_{2}}e^{-\frac{2(0.01-X+2W)}{\theta_{2}}}\times \frac{32}{\theta_{1}{}^{5}}e^{-\frac{0.31-14Y}{\theta_{1}}} \end{array}$

The second part of the likelihood, $\frac{32}{\theta_{1}{}^{5}}e^{-\frac{0.31-14Y}{\theta_{1}}}$, is bounded because *Y *< 0.015. The first part of the likelihood, $\frac{2}{\theta_{2}}e^{-\frac{2(0.01-X+2W)}{\theta_{2}}}$, goes to infinity as *X *increases towards 0.01 (but not equal to 0.01) and *W *decreases towards 0, while *θ*_2 _= 0.01-*X *+ 2*W*. Note that 0.01-*X*+2*W >0 *because *X *< 0.01 and *W *> 0. Thus we can set *θ*_2 _= 0.01-*X *+ 2*W *(all population sizes are always positive). The likelihood approaches to infinity at *θ*_2 _= 0, but because population size *θ*_2 _must be strictly positive, the MLE of the species tree for the three gene trees does not exist. Moreover, for a species tree of an arbitrary size, we can always find a rooted triple in the species tree such that the likelihood of the population indicated by the internal branch in the triple goes to infinity as the length (*W *in the previous example) of the internal branch decreases to 0 and species divergence time (*X *in the previous example) approaches to the minimum gene coalescence time (0.01 in the previous example) and thus the MLE of the species tree under the likelihood function *f*(**G**|S) does not exist. For this reason, we develop a pseudo-likelihood approach for estimating species trees from gene tree topologies. As we describe below, our method delivers MLEs for species trees, yet it is a pseudo-likelihood approach because the likelihoods of different rooted triples in the gene trees are not independent of one another. Nonetheless, we show that this method yields robust results that account for gene tree heterogeneity.

## Methods

The arguments in the previous section imply that the Rannala and Yang formula *f*(**G**|*S*) can go to infinity as we change the values of branch length *τ *and population size *θ*. To overcome this problem, the species tree *S *is reparameterized such that branch lengths are measured in coalescent units, *T = 2τ/θ *[27,37]. For the rest of this paper, branch lengths in the species tree are in coalescent units unless otherwise noted. We here develop a pseudo-likelihood to estimate the reparameterized species tree S* using topologies of gene trees. Since this method involves only topologies of gene trees, the term gene tree will be used to refer to the topology of the gene tree (without branch lengths) unless otherwise noted. The construction of the pseudo-likelihood is based on the fact that a species tree is characterized by a set of rooted triples for all subsets of three taxa [38]. Thus, estimating species tree S* is equivalent to estimating the set of rooted triples derived from S*. It motivates the rooted triple consensus method, an approach that utilizes rooted triples in gene trees to estimate the topology of the species tree [12,13,15]. Following the suggestion of Degnan et al. [39], we develop a pseudo-likelihood approach to estimate the species tree S*, including the topology and branch lengths, from a set of gene trees.

Throughout we assume that the species tree and gene trees are rooted trees. It is also assumed that the topologies of gene trees are known without error, although we show how to incorporate uncertainty in gene tree estimation into the estimate of the species tree. We assume that a single lineage is sampled from each species, as is common in phylogenetic studies [1,40]. Since coalescence occurs for at least two lineages in a population in the species tree, the lengths of the external branches (terminal populations) in the species tree are not estimable for the case of single lineage per species. Missing lineages in some gene trees are allowed if lineages are missing randomly, but a lot of missing lineages may dramatically reduce the performance of the pseudo-likelihood approach. For simplicity, we assume no missing lineages in gene trees. The theory developed in this paper can be easily extended to the cases where lineages in gene trees are missing randomly.

### Pseudo-likelihood of the Specie Tree

**Figure 2:**
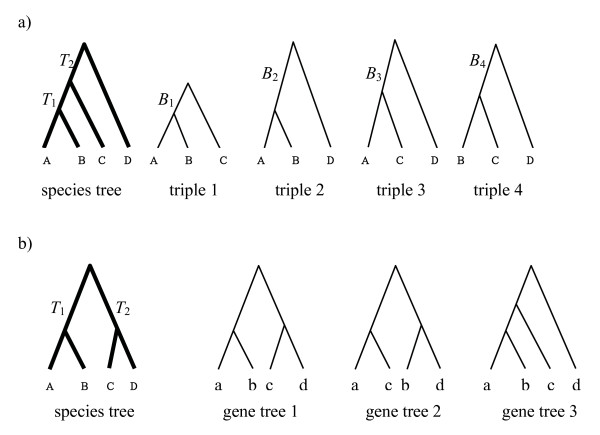
**The pseudo-likelihood of a four-taxon species tree**. a) The rooted triples of a four-taxon species tree. There are four rooted triples in the four-taxon species tree. The lengths of two internal branches in the species tree are *T*_1 _and *T*_2_. The lengths of internal branches in the four triples are *B*_1_=*T*_1_, *B*_2_=*T*_1_+ *T*_2_, and *B*_3_=*B*_4_=*T*_2_. b) The pseudo-likelihood of a four-taxon species tree. The species tree (bold lines) has two internal branches with lengths of *T*_1 _and *T*_2_. The dataset contains three gene trees (thin lines). Different gene tree triples correspond to different species tree triples. For example, triple ab|c in gene tree 1, ac|b in gene tree 2, and ab|c in gene tree 3 correspond to triple AB|C with internal branch length *B*_1 _= *T*_1 _in the species tree, while triple cd|a in gene tree 1, ac|d in gene tree 2, and ac|d in gene tree 3 correspond to triple CD|A with internal branch length *B*_2 _*= T*_2 _in the species tree.

Let *N *be the number of taxa and {*T_i_, i = 1,...*,(*N-2*)} be the lengths of internal branches in the species tree. An *N*-taxon species tree contains $\left(\begin{array}{l} N\\ 3 \end{array}\right)$ rooted triples. For example, a four-taxon species tree contains $\left(\begin{array}{l} 4\\ 3 \end{array}\right)=4$ rooted triples (Figure 2a). Let $\left\{RT_{j}{}^{S*},j=1,...,\left(\begin{array}{l} N\\ 3 \end{array}\right)\right\}$be the set of rooted triples in an *N*-taxon species tree S*. We use AB|C to denote the triple in which A and B are grouped (the first triple in Figure 2a). Each triple has an internal branch with length *B_j _*which is the sum of one or several internal branches in the species tree. In Figure 2a, the lengths of internal branches in the four triples are *B_1 _= T*_1_, *B_2 _= T*_1 _+*T_2_*, *B_3 _= B_4 _= T*_2_. Length *T*_1 _is involved in triples AB|C and AB|D, while *T*_2 _is involved in triples AC|D and BC|D. In general, an internal branch in the species tree is involved in several rooted triples in the species tree. Consider an arbitrary rooted triple $RT_{j}{}^{S^{*}}$= AB|C in the species tree and the length of the internal branch of $RT_{j}{}^{S^{*}}$ is *B_j_*. Let a, b, and c be the alleles sampled from species A, B, and C. Under coalescent, the probability that triple ab|c occurs in a gene tree randomly generated from species tree S* is $1-\left(2/3\right)e^{-B_{j}}$, whereas the probability is $\left(1/3\right)e^{-B_{j}}$ for triple ac|b or bc|a [41]. It indicates that the length *B_j _*of the internal branch of a species tree triple AB|C can be estimated by the proportion of gene trees containing triple ab|c. When the proportions of gene trees containing triples ab|c, ac|b, and bc|a are all equal to 1/3, it implies that the length of the internal branch of triple AB|C in the species tree is 0. Thus the method we develop here can be used to estimate trifurcations and polytomies in the species tree. Let *x*_*j*1_, *x*_*j*2_, and *x*_*j*3 _be the counts of triples ab|c, ac|b, bc|a occurring in gene trees. It follows that *x*_*j*1_, *x*_*j*2_, and *x*_*j*3 _have a multinomial distribution

(5) $$f\left(x_{j1},x_{j2},x_{j3}|RT_{j}{}^{S^{*}}\right)=\frac{M!}{x_{j1}!x_{j2}!x_{j3}!}{\left(1-\left(2/3\right)e^{-B_{j}}\right)}^{x_{j1}}{\left(\left(1/3\right)e^{-B_{j}}\right)}^{x_{j2}}{\left(\left(1/3\right)e^{-B_{j}}\right)}^{x_{j3}}$$

where *M *is the sum of *x*_*j*1_, *x*_*j*2_, and *x*_*j*3_. Note that *M *is equal to the number of genes (loci) for all *j*. The MLE of $RT_{j}{}^{S^{*}}$ under (5) is the most frequent triple among ab|c, ac|b, and bc|a occurring in gene trees with the length of the internal branch

(6) $${\hat{B}}_{j}=-\log\{3\times (1-x/M)/2\}\;\text{for}\;x\neq M.$$

Here *x *is the count of the most frequent triple among ab|c, ac|b, and bc|a occurring in gene trees. Note that *x *>*M*/3 and thus ${\hat{B}}_{j}$ is positive. Because the proportion *x*/*M *in (6) converges to its expectation $1-(2/3)e^{-B_{j}}$, the MLE ${\hat{B}}_{j}$ converges to the true length *B_j _*in probability, i.e.,

(7) $${\hat{B}}_{j}=-\log\left\{3\times (1-x/M)/2\right\}\overset{p}{\to }B_{j},$$

as *M *goes to infinity. This indicates that the frequencies of triples in gene trees can be used to consistently estimate both topologies and internal branch lengths (in coalescent units) of triples in the species tree. The joint probability distribution of triples in the species tree is approximated by the product of marginal probabilities (thus the name of pseudo-likelihood) [42,43]. Although the approximation ignores the correlation structure among interrelated triples in gene trees (and thus does not utilize all the information in the data) when estimating species trees, the pseudo-likelihood has computational advantages as the joint probability distribution of triples is difficult to calculate and the MLE under the full likelihood function (or the Rannala and Yang formula) does not exist. In addition, we can show (in the next section) that the estimate of the species tree obtained by maximizing the pseudo-likelihood is statistically consistent. The pseudo-likelihood of species tree S* given gene trees **G **is defined as the product of the multinomial distributions in (5) across all triples in the species tree:

(8) $$L(S*|\text{G})=w\times \prod_{j=1}^{\left(\begin{array}{l} N\\ 3 \end{array}\right)}\left\{{\left(1-(2/3)e^{-B_{j}}\right)}^{x_{j1}}{\left((1/3)e^{-B_{j}}\right)}^{x_{j2}}{\left((1/3)e^{-B_{j}}\right)}^{x_{j3}}\right\}$$

where $w=\prod_{j=1}^{\left(\begin{array}{l} N\\ 3 \end{array}\right)}\left\{\frac{M!}{x_{j1}!x_{j2}!x_{j3}!}\right\}$, *B_j _*is the length of the internal branch of triple *j *in the species tree, and *x*_*j*1_, *x*_*j*2_, and *x*_*j*3 _are the frequencies of three types of triples in gene trees. The estimate of the species tree, including topology and internal branch lengths, is obtained by maximizing the pseudo-likelihood *L*(S*|**G**). Since *w *is a function of *x *and has no effect on the procedure of maximizing *L*(S*|**G**), it can be ignored from the likelihood function *L*(S*|**G**). We employ a heuristic search technique; nearest-neighbor interchanges (NNI), to find the maximum pseudo-likelihood estimate (MPE) of the species tree. We call this method Maximum Pseudo-likelihood for Estimating Species Trees, or MP-EST. When *N *= 3, (8) becomes (5) and the MP-EST tree is the most frequent gene tree triple with an internal branch of length described in (6). Note that the length *B_j _*of the internal branch of triple *j *in the species tree is the sum of one or several internal branch lengths {*T_i_*, *i *= 1,...,(*N*-1)} in the species tree. Because the length *T_i _*of internal branch *i *in the species tree involves in many species tree triples, it is estimated by the combination of the frequencies of the gene tree triples corresponding to the species tree triples that involve *T_i_*. Equation (6) implies that the estimate ${\hat{B}}_{j}$ of the length of the internal branch in the species tree triple $RT_{j}{}^{S^{*}}$ increases to infinity as the proportion *x/M *of the most frequent gene tree triple approaches to 1. Thus the length of an internal branch in the species tree is not estimable if all relevant triples in gene trees support the same topology. In this case, we assign "99" as the length of the branch to indicate that this branch length is not estimable.

As an example, we calculate the pseudo-likelihood for a four-taxon species tree with a fixed topology and two internal branches of lengths *T*_1 _and *T*_2 _(Figure 2b). There are four rooted triples for this four-taxon species tree. Let *B*_1_, *B*_2_, *B*_3_, and *B*_4 _be the lengths of internal branches in triples AB|C, AB|D, CD|A, CD|B. It follows that *B*_1 _= *B*_2 _= *T*_1 _and *B*_3 _= *B*_4 _= *T*_2_. Suppose that the dataset contains three gene trees (Figure 2b). To calculate the pseudo-likelihood in (8), we need to count the numbers of gene tree triples corresponding to each of the four species tree triples. There are 2 ab|c triples, 1 ac|b triple, and 0 cb|a triple in gene trees corresponding to triple AB|C in the species tree. The likelihood of triple AB|C with internal branch length *B*_1 _= *T*_1 _in (8) is

$${\left(1-\left(2/3\right)e^{-T_{1}}\right)}^{2}{\left(\left(1/3\right)e^{-T_{1}}\right)}^{1}{\left(\left(1/3\right)e^{-T_{1}}\right)}^{0},$$

Similarly, we can calculate the likelihoods of the other three triples in the species tree.

The pseudo-likelihood of the species tree for this dataset is equal to

$$\begin{array}{l} \overset{\text{triple AB|C}}{\overset{︷}{{\left(1-\left(2/3\right)e^{-T_{1}}\right)}^{2}{\left(\left(1/3\right)e^{-T_{1}}\right)}^{1}{\left(\left(1/3\right)e^{-T_{1}}\right)}^{0}}}\times \overset{\text{triple AB|D}}{\overset{︷}{{\left(1-\left(2/3\right)e^{-\left(T_{1}\right)}\right)}^{2}{\left(\left(1/3\right)e^{-\left(T_{1}\right)}\right)}^{1}{\left(\left(1/3\right)e^{-\left(T_{1}\right)}\right)}^{0}}}\times \\ \underset{\text{triple CD|A}}{\underset{︸}{{\left(1-\left(2/3\right)e^{-T_{2}}\right)}^{1}{\left(\left(1/3\right)e^{-T_{2}}\right)}^{2}{\left(\left(1/3\right)e^{-T_{2}}\right)}^{0}}}\times \underset{\text{triple CD|B}}{\underset{︸}{{\left(1-\left(2/3\right)e^{-T_{2}}\right)}^{1}{\left(\left(1/3\right)e^{-T_{2}}\right)}^{1}{\left(\left(1/3\right)e^{-T_{2}}\right)}^{1}}} \end{array}$$

The estimates of the lengths of internal branches in the species tree are obtained by maximizing the pseudo-likelihood with respect to *T*_1 _and *T*_2_. Note that *T*_1 _involves in the likelihoods of two triples AB|C, AB|D, while *T*_2 _involves in the likelihoods of CD|A and CD|B. Thus *T*_1 _is estimated by the frequencies of gene tree triples corresponding to the species tree triples AB|C and AB|D and *T*_2 _is estimated by the frequencies of gene tree triples corresponding to the species tree triples CD|A and CD|B. Due to the simplicity of this example, we can explicitly derive the estimates of *T*_1 _and *T*_2_,

$$\begin{array}{l} {\hat{T}}_{1}=-\log\left\{\frac{3}{2}(1-\frac{\left(y_{1}+y_{2}\right)}{2k})\right\}=-\log\left\{\frac{3}{2}\left(1-\frac{\left(2+2\right)}{6}\right)\right\}=0.693\\ {\hat{T}}_{2}=-\log\left\{\frac{3}{2}\left(1-\frac{\left(y_{3}+y_{4}\right)}{2k}\right)\right\}=-\log\left\{\frac{3}{2}\left(1-\frac{\left(1+1\right)}{6}\right)\right\}=0 \end{array}$$

where *y*_1_, *y*_2_, *y*_3 _and *y*_4 _are the counts of gene tree triples ab|c and ab|d, cd|a, and cd|b. The estimate of *T*_2 _is 0 because the proportions of gene tree triples cd|a and cd|b matching the species tree triples CD|A and CD|B are equal to 1/3. The log-likelihood of the species tree with *T*_1 _= 0.693 and *T*_2 _= 0 is -11.797. There are 15 possible topologies for a four-taxon species tree. Similarly, we can calculate the log-likelihoods for the other 14 topologies and choose the one with the maximum log-likelihood score as the estimate of the species tree (topology and branch lengths).

### Statistical Consistency

We use Φ(S*) to denote the pseudo-likelihood of a species tree S* in (8) without *w *because *w *has no effect on the procedure of maximizing *L*(S* | **G**).

(9) $$\Phi \left(S^{*}|\text{G}\right)=\prod_{j=1}^{\left(\begin{array}{l} N\\ 3 \end{array}\right)}\left\{{\left(1-\left(2/3\right)e^{-B_{j}}\right)}^{x_{j1}}{\left(\left(1/3\right)e^{-B_{j}}\right)}^{x_{j2}}{\left(\left(1/3\right)e^{-B_{j}}\right)}^{x_{j3}}\right\}$$

The MPE of the species tree is ${\hat{S}}^{*}=\underset{S^{*}}{\arg\max}\left\{\Phi \left(S^{*}\right)\right\}$. It follows from the strong law of large numbers [44] that as the number of genes *M *increases to infinity, the proportions of triples in gene trees converge to their expectations almost surely,

(10) $$\left\{\frac{x_{j1}}{M},\;\frac{x_{j2}}{M},\;\frac{x_{j3}}{M}\right\}\overset{a.s}{\to }\left\{\;(1-(2/3)e^{-W_{j}}),\;((1/3)e^{-W_{j}}),\;((1/3)e^{-W_{j}})\;\right\}\text{ for }j=1,...,\left(\begin{array}{l} N\\ 3 \end{array}\right).$$

where *W*_j _is the length of the internal branch of triple *j *in the true species tree *S_T_*. Thus as *M *→ ∞, Φ (S* | *G*) converges to function H(S*) almost surely,

(11) $$Η\left(S^{*}\right)=\prod_{j=1}^{\left(\begin{array}{l} N\\ 3 \end{array}\right)}\left\{\;{\left(1-\left(2/3\right)e^{-B_{j}}\right)}^{M\left(1-\left(2/3\right)e^{-W_{j}}\right)}{\left(\left(1/3\right)e^{-B_{j}}\right)}^{M\left(1/3\right)e^{-W_{j}}}{\left(\left(1/3\right)e^{-B_{j}}\right)}^{M\left(1/3\right)e^{-W_{j}}}\right\}$$

Because Φ (S* | *G*) and H(S*) are bounded continuous functions (0 < Φ (S* | *G*) < 1 and 0< H(S*) < 1). In addition, the function H(S*) is maximized when *B*_j _= *W*_j _for all *j*, i.e., $S_{T}=\underset{S^{*}}{\arg\max}\left\{Η\left(S^{*}\right)\right\}$. Thus as *M *→ ∞, the MPE ${\hat{S}}^{*}$ converges to the true species tree *S_T _*in probability, i.e.,

(12) $${\hat{S}}^{*}\overset{p}{\to }S_{T}\text{,}$$

which shows that the MP-EST method is statistically consistent in estimating species trees (topology and branch lengths in coalescent units) as the number of genes increases.

We next derive the rate at which the probability $P\left\{{\hat{S}}^{*}\overset{top}{=}S_{T}\right\}$ that the MPE ${\hat{S}}^{*}$ is topologically identical to the true species tree *S_T _*converges to 1. Equations (10), (11), and (12) imply that the MPE ${\hat{S}}^{*}$ matches the true species tree *S_T _*in topology if all the differences between the proportions of the most frequent gene tree triples and their expectations are very small, i.e.,

(13) $$\left|\frac{x_{j1}}{M}-\left(1-\left(2/3\right)\;e^{-W_{j}}\right)\right|<\varepsilon \;\text{for }j=1,...,\left(\begin{array}{l} N\\ 3 \end{array}\right),$$

where *ε *is a small positive real number determined by the true species tree *S_T_*. Thus,

(14) $$\begin{array}{c} P\left\{{\hat{S}}^{*}\overset{top}{=}S_{T}\right\}>P\left[\;\left|\;\frac{x_{j1}}{M}-\left(1-\left(2/3\right)e^{-W_{j}}\right)\;\right|<\varepsilon ,\text{for each j}\in \left\{1,...,\left(\begin{array}{l} N\\ 3 \end{array}\right)\right\}\right]\\ >1-\sum_{j=1}^{\left(\begin{array}{l} N\\ 3 \end{array}\right)}P\left\{\;\left|\;\frac{x_{j1}}{M}-(1-(2/3)e^{-W_{j}})\;\right|\geq \varepsilon \right\}\\ >1-\sum_{j=1}^{\left(\begin{array}{l} N\\ 3 \end{array}\right)}\frac{\mathrm{var}(x_{j1}/M)}{\varepsilon^{2}}=1-\sum_{j=1}^{\left(\begin{array}{l} N\\ 3 \end{array}\right)}\frac{\left(2/3)e^{-W_{j}}\times (1-(2/3)e^{-W_{j}}\right)}{M\varepsilon^{2}}\\ >1-\frac{\left(\begin{array}{l} N\\ 3 \end{array}\right)}{4M\varepsilon^{2}} \end{array}$$

This shows that the probability of ${\hat{S}}^{*}$ matching the true species tree *S_T _*converges to 1 at *O*(*M*^-1^).

### Robustness to Horizontal Gene Transfer (HGT)

**Figure 3:**
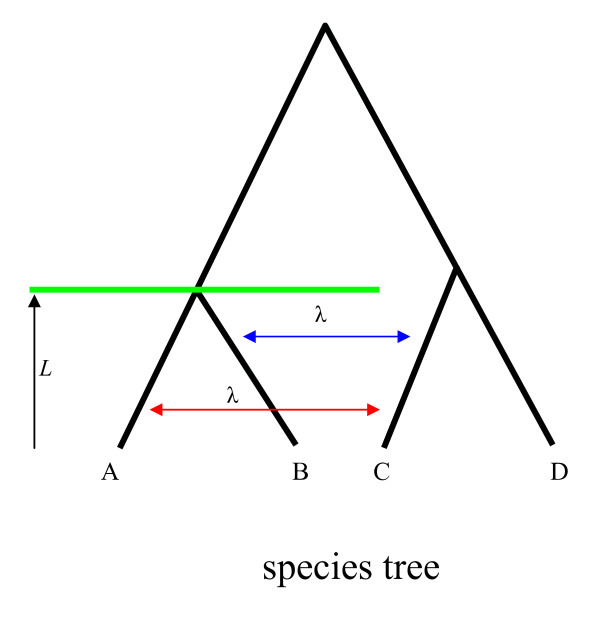
**Horizontal gene transfer (HGT) events in the species tree**. The species tree is an ultrametric tree. Branch lengths in the species tree are the number of generations. We here focus on the HGT events that can change the topology of species A, B, and C. HGT events between species A and C (red arrow) or between species B and C (blue arrow) occurring below the divergence time (green line) of species A and B can change the topology of species A, B, and C. We assume that the transfer rate between species A and C (or between species B and C) is λ (per generation). Thus the probability distribution of the number of HGT events occurring between species A and C or species B and C below the divergence time *L *has a Poisson distribution with mean 2λ*L*.

Consider an arbitrary triple AB|C in the species tree and the length of the internal branch is *B_j_*. Under coalescent, the probability that a triple in the gene trees generated from the species tree topologically agrees with triple AB|C is $1-(2/3)e^{-B_{j}}$. Although HGT events may occur between species A and B, species A and C, or species B and C, we here only consider the HGT events between species A and C, or B and C because these events can change the probability of observing a matching triple in gene trees and may thus result in a biased MP-EST estimate of the species tree. Suppose that the rate of horizontal gene transfer λ is homogeneous per gene per generation between extant species A and C (or B and C) in triple AB|C (Figure 3). We assume that the probability distribution of the number of HGT events occurring between two species is a Poisson distribution with mean λ*L *where *L *is the divergence time (in generations) of two species. The probability that HGT events occur between species A and C or species B and C is 1-e^-2λL ^(Figure 3). Adding HGT events in the coalescent model, the probability of observing triple ab|c in gene trees becomes

(15) $$1-\left(2/3\right)e^{-B_{j}}-\left(1-e^{-2\lambda L}\right)=e^{-2\lambda L}-\left(2/3\right)e^{-B_{j}}.$$

It follows that the proportion of gene trees containing triple ab|c converges to the quantity specified in (15) and the MP-EST estimate ${\hat{B}}_{j}$ of the length of the internal branch in triple AB|C does not converge to the true length *B_j _*when the rate λ of HGT is not 0. It implies that some estimates ${\hat{T}}_{i}$s of the lengths of internal branches in the species tree do not converge to the true lengths *T_i _*s. Otherwise if all estimated lengths ${\hat{T}}_{i}$s converge to the true lengths *T_i _*s, then all ${\hat{B}}_{j}$s will converge to *B_j _*s (because *B_j _*s are the sums of one or several *T_i _*s), which contradicts the previous conclusion that the MP-EST estimate ${\hat{B}}_{j}$ of the length of the internal branch in triple AB|C does not converge to the true length *B_j_*. Inconsistency of the MP-EST estimates ${\hat{T}}_{i}$s of the lengths of internal branches in the species tree is due to the HGT events occurring among species A, B, and C. Because an internal branch in the species tree is estimated by the combination of the proportions of the gene tree triples corresponding to the species tree triples that involve this branch, the effect of the biased proportion of gene tree triples (due to the HGT events occurring among species A, B, and C) on the estimation of branch lengths is alleviated by the proportions of other gene tree triples that are not affected by HGT, especially when HGT events only occur in a small group of species.

Although HGT events can result in biased estimates of the lengths of internal branches in the species tree, the topology of the species tree may still be consistently estimated when *λ *is small. Previously, we have shown that the topology of the species tree can be consistently estimated if the conditions in (13) hold for some large *M*. Thus we want to find a large *M *so that

(16) $$\;\left|x_{j1}/M-\left(1-\left(2/3\right)e^{-W_{j}}\right)\right|<\varepsilon ,$$

where *x*_*j*1 _is the count of the most frequent gene tree triple. We know that the proportion *x*_*j*1_/*M *converges to $e^{-2\lambda L}-(2/3)e^{-B_{j}}$ (equation (15)) when HGT occurs at rate *λ*. It implies that for any δ > 0, there exists a large *M *such that $\left|x_{j1}/M-\left(1-(2/3)e^{-W_{j}}\right)+1-e^{-2\lambda L}\right|<\delta$, i.e., $\left|x_{j1}/M-\left(1-\left(2/3\right)e^{-W_{j}}\right)\;\right|<\delta +1-e^{-2\lambda L}$. Thus (16) holds if 1-*e*^-2λ*L *^< ε because *α *becomes very small and negligible when *M *is large. We conclude that (16) holds if $\lambda <\frac{-\log\left(1-\varepsilon \right)}{2L}$. It shows that if the rate (*λ*) of horizontal gene transfer is smaller than $\frac{-\log\left(1-\varepsilon \right)}{2L}$, the MP-EST method is still statistically consistent in estimating the topology of the species tree.

### Estimating species trees from multilocus sequences

The proof for the consistency of the MP-EST method is based on the assumption that gene trees are known without error. In fact, gene trees are usually unknown and must be estimated from multilocus sequences. Under general conditions, the ML gene tree $\hat{G}$ estimated from sequence data is a consistent estimator of the true gene tree *G *[45]. Thus the MPE of the species tree ${\tilde{S}}^{*}$ based on the estimated gene tree is a consistent estimator of the species tree S*. The procedure of estimating species trees from multilocus sequences includes two steps; gene trees are first independently estimated from mutlilocus sequences using the ML method [46] or any other method that is consistent, and rooted by an outgroup (we assume that the outgroup is known). Although gene trees can be rooted by multiple outgroups, it requires that the outgroup sequences must form a monophyletic clade consistently in all gene trees, which rarely occurs in reality. Thus when the appropriate outgroup includes multiple species and is comprised of a monophyletic group, then one must drop some sequences in order to estimate gene trees with a single sequence as the outgroup. However, multiple outgroups can be accommodated partially if they do not comprise a monophyletic group. In this case a single outgroup sequence is again used, and the additional outgroups are estimated as if they were part of the ingroup. Multiple species cannot simultaneously be designated as outgroups (for rooting gene trees) using MP-EST.

The rooted gene trees are then used to construct the MP-EST tree as the estimate of the species tree. Although ML gene trees are usually binary trees, there may be some cases in which some internal nodes and relevant rooted triples in gene trees are unresolved (polytomy). For an unresolved triple (a,b,c) in gene trees, we assign 1/3 to each of the three possible topologies ab|c, ac|b, and bc|a. The estimation error in gene trees can be incorporated into the analysis using bootstrapping techniques [47,48]. Specifically, columns (or sites) in the aligned sequences are resampled with replacement for each gene sampled (with replacement) from the multilocus dataset [48,49]. The MP-EST trees constructed for the bootstrapped samples are summarized by a consensus tree.

## Results

### Simulation

To evaluate the performance of MP-EST, we simulated 10 ten-taxon species trees from a birth and death process with birth rate of 10 and death rate of 0.1 from the phylogenetic program Mesquite [50]. The population sizes (θ) in the species tree were generated from a uniform distribution (0.005, 0.01). Branch lengths in the 10 species trees vary in the range of 0.00029 and 0.01832. To convert it to coalescent units, the branch length must be divided by the population size *θ*. Gene trees were generated from the ten species trees using the phylogenetic program Phybase [51] and then used as data to estimate species trees by MP-EST, STAR [11], and Rooted Triple consensus (RT) [12]. We repeated the simulation 100 times. The performance of each method was evaluated based on the proportion of trials yielding the true species trees. We also evaluated the mean square error (MSE) [52] of the branch length between the true species tree and the MP-EST estimate of the species tree. When calculating the MSE of the branch length, we discarded the branches with length "99" because the lengths of these branches are not estimable.

**Figure 4:**
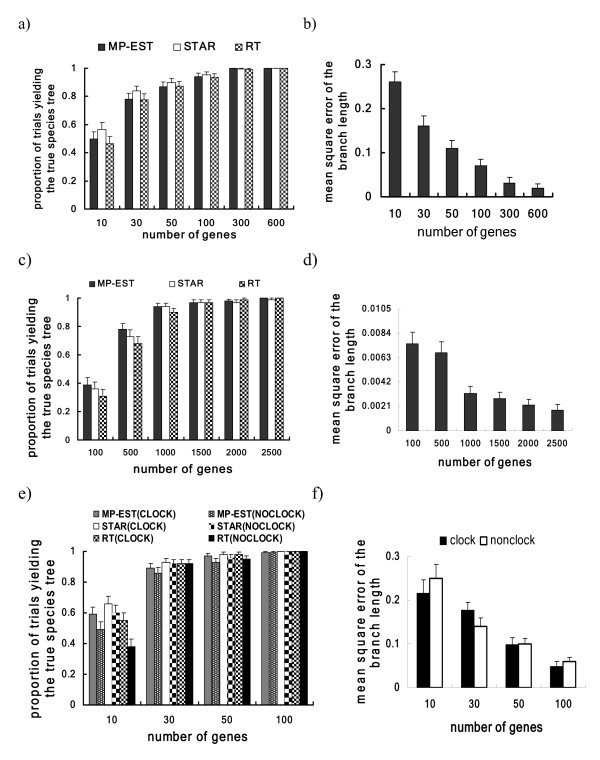
**The performance of MP-EST in estimating species trees**. (a, b) the performance of MP-EST, STAR, and RT in estimating species trees from gene trees. (c, d) the performance of MP-EST, STAR, and RT in estimating an anomalous species tree. (e, f) the performance of MP-EST, STAR, and RT in estimating species trees from DNA sequences. Because STAR and RT cannot estimate branch lengths of the species tree, the results in (b,d,f) do not include STAR and RT.

The results (Figure 4a and 4b) suggest that as the number of genes increases, the proportion of trials in which MP-EST has successfully recovered the true species trees increases to 1, and the MSE of the branch length (in coalescent units) appears to decrease to 0, indicating that MP-EST is statistically consistent in estimating the species trees (topology and branch length in coalescent units) generated in the simulation. The results for STAR and RT show the same pattern that as the number of genes increases, the proportions of trials yielding the true species tree for both methods increase to 1. Overall, STAR performs slightly better than the other two methods, while MP-EST and RT have the similar performance in recovering the true species tree (Figure 4a). A low proportion in Figure 4a does not necessarily imply a large topological difference between the true species tree and the tree estimated by a species tree reconstruction method. For example, the proportion of the MP-EST trees matching the true species tree is 0.47 for the case of 10 genes (Figure 4a), but across all replicates the average Robinson and Foulds (RF) topological distance [53] between the MP-EST tree and the true species tree is 1.35, indicating that on average only one or two internodes (usually with short branches leading to their ancestral nodes) are not successfully recovered.

In the second simulation, we investigate the performance of MP-EST, STAR, and RT in estimating species trees in the anomaly zone. The anomaly zone is a region of species tree space, one with very short branches in the species tree, in which the most common gene tree is different from the species tree [37]. One would not expect estimation of species trees in this case to be straightforward. Gene trees were simulated from an anomalous species tree ((((A:0.5, B:0.5):0.025, C:0.525):0.025, D:0.55):1, E:1.55) (in coalescence units). The most probable gene tree has the topology (((A,B),(C,D)),E) and the RF distance between the true species tree and the most probable gene tree is 2. The generated gene trees were then used as data to infer the species tree using MP-EST, STAR, and RT. The simulation was repeated 100 times and we calculated the proportion of trials yielding the true species tree for each species tree reconstruction method. We also calculated the MSE of the branch length between the MP-EST tree and the true species tree. The result for the MP-EST method shows that the proportion of trials yielding the true species tree increases to 1, while the MSE of the branch length appears to decrease to 0, as the number of genes increases (Figure 4c and 4d). This confirms that MP-EST can consistently estimate the true species tree even in the anomaly zone, as expected from the theory we developed above. Similarly, the proportions of the STAR and RT trees matching the true species tree approach 1 as the number of genes increases. In this simulation, MP-EST appears to outperform STAR and RT at 100 and 500 genes (Figure 4c). In addition, the result suggests that all three methods require a large number of genes to accurately estimate anomalous species trees (Figure 4c).

We next investigated estimation of species trees from alignments of DNA sequences. In this simulation, a species tree was generated from a birth and death process: (A:0.019, (((B:0.01, C:0.01):0.0017, ((D:0.00003, E:0.00003): 0.00666, F:0.0067):0.005004):0.00312,((G:0.0043,(H:0.0003,I:0.0003):0.004):0.0034,J:0.0077):0.007):0.0038) with population sizes generated from a uniform distribution (0.005, 0.01). The branches in the species tree are in mutation units. Gene trees were generated from this species tree assuming a molecular clock and then used to simulate DNA sequences of 500 bp under the Jukes-Cantor model in Phybase [51]. The average height and branch length of the simulated gene trees are 0.0243 and 0.0072 in substitutions per site. Another set of DNA sequences were simulated from the gene trees generated from a non-clocklike species tree model which assumes that the substitution rate is the same for all genes and sequences in the same population in the species tree, but the rates may differ across populations [6]. The terminal and internal branches (terminal and ancestral populations) of the species tree were assigned with relative mutation rates generated from a Dirichlet distribution with the shape parameter *β *= 0. The branches of the gene tree entering a particular population in the species tree are multiplied by the relative mutation rate of that population. The simulated DNA sequences were used to estimate the species tree. ML gene trees were first estimated independently and without a molecular clock for each gene in the phylogenetic program PHYML [54] with the Jukes-Cantor model (we used the default for other parameters in PHYML) and rooted by species A. The MP-EST, STAR, and RT trees were constructed from the estimated gene trees. The simulation was repeated 100 times. For the MP-EST method, the proportion of trials yielding the true species tree appears to approach 1, while the MSE of the branch length goes towards 0, as the number of genes increases (Figure 4e and 4f), which suggests that MP-EST is statistically consistent in estimating species trees from multilocus sequences, not just when gene trees are given as in the first simulation. Overall, STAR slightly outperforms MP-EST and RT (Figure 4e). In addition, STAR, MP-EST, and RT perform better for the sequences generated from the clocklike species tree than those generated from the non-clocklike species tree, especially when the number of genes is small (10 genes in Figure 4e). Nevertheless, the proportions of STAR, MP-EST, and RT trees matching the true species tree increase to 1 as the number of genes increases to 100, regardless the sequences were generated from a clocklike species tree or a non-clocklike species tree. It suggests that MP-EST, STAR, and RT can consistently estimate the species tree in the absence of a molecular clock. The robustness of the methods to violating the clock is due to the fact that all three methods use only the topologies of gene trees to estimate species trees. In this simulation, we have demonstrated that MP-EST is statistically consistent for the cases of violating the clock randomly throughout the species tree and gene trees. There might be other ways of violating the clock for which MP-EST may not be consistent. For instance in long-branch attraction types of scenarios where gene trees can not be consistently estimated from molecular sequences, MP-EST may consistently estimate a wrong species tree due to the bias in the gene tree reconstruction. We observed that nearly half (49%) of the gene trees generated from the species tree across replicates were rooted incorrectly and species A was not located on the branch between (BCDEF) and (GHIJ). Yet in these cases and in the simulation generally, the correct species tree was always consistently estimated. This result suggests that MP-EST (STAR and RT) can consistently recover the true species tree even when estimated gene trees are misrooted frequently. The theory we developed assumes that the roots of the gene trees are known without error, yet our simulation suggests that this assumption can be violated and still yield a solid result under the coalescent model.

**Figure 5:**
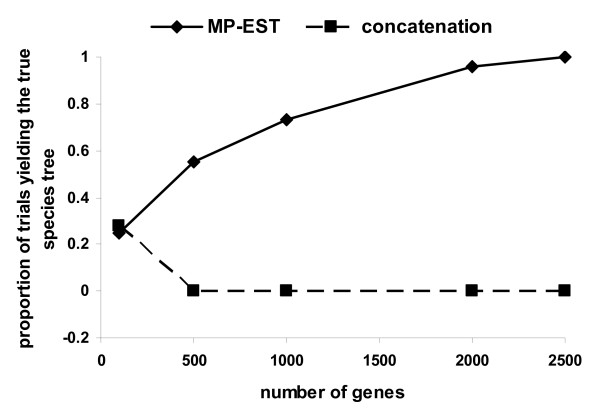
**Comparison between MP-EST and the Bayesian concatenation method in estimating an anomalous species tree**. DNA sequences were simulated from an anomalous species tree and used as data to estimate the species tree by the MP-EST and Bayesian concatenation methods. The simulation was repeated 100 times and we calculated the proportion of trials yielding the true species tree for each of the two species tree reconstruction methods.

Concatenation approaches have been frequently used to infer species trees from multilocus sequences [4,40,55]. To compare MP-EST with concatenation, we simulated DNA sequences from an anomalous species tree ((((A:0.005, B:0.005):0.00025, C:0.00525):0.00025, D:0.0055):0.01, E:0.0155) with a constant population size *θ *= 0.01 for all populations in the tree. The lengths of branches are in mutation units. Species E is used as the outgroup to root gene trees in the MP-EST analysis. Gene trees were generated from this species tree assuming a molecular clock and then used to simulate DNA sequences of 500 bp under the Jukes-Cantor model in Phybase. Species trees were estimated from the simulated sequences using the MP-EST and Bayesian concatenation methods. For the Bayesian concatenation method, the species tree was estimated by the consensus tree constructed from the posterior distribution of the species tree estimated in the Bayesian phylogenetic program MrBayes [56] with the Jukes-Cantor model. The chains (one cold chain and three hot chains) ran for 1000000 generations and we saved every 100^th ^trees after a burnin period of 500000 generations. The simulation and Baysian concatenation analysis were repeated 100 times. We selected a sample of simulation repetitions to check convergence of the MCMC algorithm and found that all MCMC algorithms converged at the 10000th generation (the standard deviation of split frequencies < 0.0001). MP-EST trees were constructed as described in the previous simulations. The result for the MP-PEST method shows that the proportion of trials yielding the true species tree approaches 1.0 as the number of genes increases (Figure 5). In contrast, the proportion of the concatenation trees matching the true species tree goes to 0 (Figure 5). This result suggests that MP-PEST outperforms the Bayesian concatenation method in the anomaly zone.

Although all results suggest that MP-EST is statistically consistent in estimating species trees, the ranges of genes required for MP-EST to recover the true species tree with a high probability are largely different across simulations. In the first simulation (Figure 4a), it requires at least 100 genes for the proportion of trials yielding the true species tree to reach 0.9. It increases to 1000 genes in the second simulation (Figure 4c), but decreases to 50 genes in the third simulation (Figure 4e). The number of genes needed depends on the true species tree. In general, it requires more genes to accurately estimate the species tree with short internal branches (in coalescent units) than to accurately estimate the species tree with long internal branches (in coalescent units). This explains why it needs a large number of genes in the second simulation where the species tree is in the anomaly zone and has very short internal branches (in coalescent units).

### Mammal data analysis

**Figure 6:**
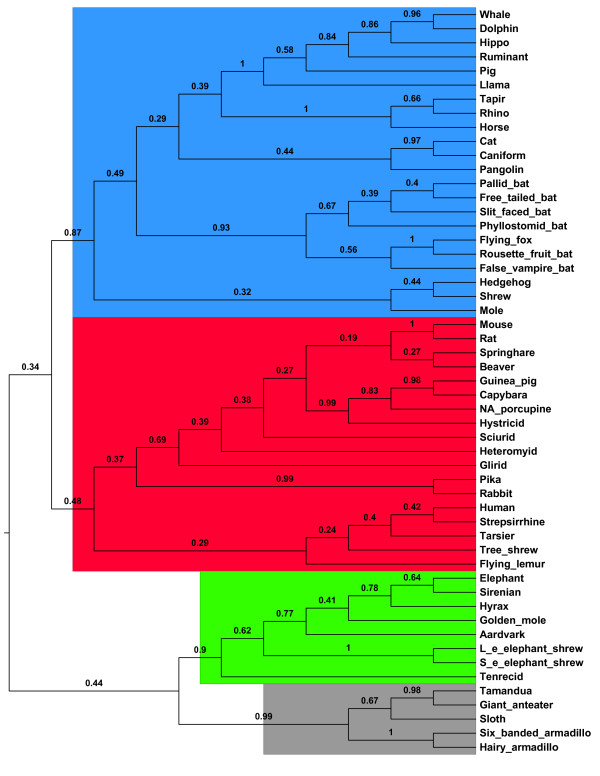
**The MP-EST consensus tree for the mammal dataset**. A consensus tree was constructed from the species trees estimated by the MP-EST method for 1000 bootstrap datasets using Majority-Rule-extension (MRe) in CONSENSUS from the PHYLIP package. The numbers on the branches are bootstrap proportions based on 1000 replicates. There are four monophyletic clades: *Laurasiatheria *(blue), *Euarchontoglires *(red), *Xenarthra *(gray), *Afrotheria *(green). The outgroup *opossum *has been excluded.

Springer et al. [57] used mutilocus DNA sequences to estimate the phylogenetic relationship among placental mammals. The dataset contains DNA sequences from 20 genes for 53 placental mammals and 4 marsupial outgroups (*opossum*, *diprotodontian*, *monitor del monte*, *shrew opossum*), totalling 14,326 sites. Because the four outgroup sequences do not consistently form a monophyletic group for all genes, we reduced the original data set from 57 to 54 species so that a single outgroup (*opossum*) rather than multiple outgroups is included as required by MP-EST. In the MP-EST analysis, 1000 bootstrap samples were produced using a nonparametric bootstrapping technique [47]. ML gene trees were estimated for 1000 bootstrap samples in PHYML and rooted with outgroup *opossum*. The rooted ML gene trees were used to construct 1000 MP-EST trees. A consensus tree (Figure 6) was built from the 1000 MP-EST trees using Majority-Rule-extension (MRe) in CONSENSE from the PHYLIP package [58]. The MP-EST tree with branch lengths (in coalescent units) was plotted in Additional file 1: Figure S1.

Since the MP-EST method can accommodate only a single outgroup sequence (opossum), it suffers from of its inability to utilize multispecies outgroups, which are widely thought to help stabilize the roots of the estimated gene trees [59]. To investigate the problem of multispecies outgroups, we repeated the MP-EST analysis for the original mammal data set including the four marsupial outgroups (*opossum*, *diprotodontian*, *monitor del monte*, *shrew opossum*). ML gene trees were still rooted with outgroup *opossum*. The phylogenetic relationships of placental mammals in the MP-EST consensus tree for the full mammal data set are consistent with those constructed from the reduced data set (Additional file 2: Figure 2). In addition, the other 3 outgroup species (*diprotodontian*, *monitor del monte*, *shrew opossum*) in the MP-EST consensus tree form a basal clade with a high bootstrap proportion 0.98 (Additional file 2: Figure 2). However, we note that if the opossum outgroup is included, this results in a lack of monophyly for marsupials, which is clearly incorrect. Ultimately MP-EST is unable to accommodate multiple outgroups when the outgroup clade is monophyletic. Therefore we are forced to use a single outgroup and to drop other species that are more closely related to that outgroup than to the ingroup.

Unlike the highly supported Bayesian concatenation tree, most bootstrap proportions in the MP-EST consensus tree are less than 0.5 (Figure 6). It may be inappropriate to compare bootstrap supports with posterior probabilities because the relation between bootstrap values and posterior probabilities are highly variable [60,61] and no studies have been conducted to assess the correlation between bootstrap supports and posterior probabilities at the species tree level. Neither bootstrap values nor posterior probabilities are measures of accuracy of the estimated phylogenetic trees. However, separate analyses for 20 gene segments of the mammal data set produced poorly supported gene trees (Additional file 3: Figure S3). Approximately 80% of the bootstrap values in the estimated gene trees are less than 0.5. In addition, most bootstrap values for deep phylogenetics relationships are less than 0.2. The poorly supported gene trees suggest that the mammal data set does not contain much information about the phylogenetic relationship of placental mammals. Nevertheless, most posterior probabilities in the Bayesian concatenation tree are equal to 1.0, indicating that the Bayesian concatenation method may have overestimated the posterior probabilities. In contrast, the low bootstrap supports in the MP-EST consensus tree have reasonably reflected uncertainty in the estimated gene trees. There are in general two types of genetic variations among multilocus sequences; genetic variation among loci and genetic variation within each locus. In the MP-EST analysis, both variations are considered in the nonparametric bootstrap technique. As a result, the bootstrap supports in the MP-EST consensus tree reflect the level of uncertainty of clades within and among gene trees. For example, the MP-EST consensus tree for the mammal data set has high bootstrap supports for the branches close to the terminal tips and low bootstrap supports for the branches close to the tree root, which is consistent with the pattern of bootstrap values in gene trees (Additional file 3: Figure S3). In contrast, the Bayesian concatenation method assumes congruent gene trees. The spuriously high posterior probabilities, as those in the Bayesian concatenation tree for the mammal data set, are probably due to the assumption of congruent gene trees, along with the fact that bootstrap values are more conservative than posterior probabilities as the measure of the reliability of phylogenetic trees [61].

Despite the molecular and genomic consensus of the four-clade classification of placental mammals (*Xenarthra*, *Laurasiatheria*, *Euarchontoglires *and *Afrotheria*), the relationship among the four major groups is highly controversial. Three different hypotheses regarding the topology of the four clades were supported by different phylogenetic markers [62]. Morphological markers and retroposons data favored the topology (*Xenarthra*, (*Afrotheria*, (*Laurasiatheria*, *Euarchontoglires*))) [63,64]. The second topology ((*Xenarthra*, *Afrotheria*), (*Laurasiatheria*, *Euarchontoglires*)) was supported by phylogenetic studies of the full mitochondrial genome [62,65], while the analyses of protein-coding and non-coding sequences supported the topology (*Afrotheria*, (*Xenarthra*, (*Laurasiatheria*, *Euarchontoglires*))) [57,66]. More recently, the genome-wide analysis and large scale sequence data provided evidence for a clear trifurcation at the root of placentals [67,68]. Nishihara et al [69] came to the same conclusion based on the retroposon analysis and recent geological data. The ancestral relationship for the four major groups in the MP-EST consensus tree is ((*Xenarthra*, *Afrotheria*), (*Laurasiatheria*, *Euarchontoglires*)) with bootstrap support 0.47, while the second most supported relationship is (*Afrotheria*, (*Xenarthra*, (*Laurasiatheria*, *Euarchontoglires*))) with support 0.37. Moreover, the bootstrap support for the relationship (*Xenarthra*, (*Afrotheria*, (*Laurasiatheria*, *Euarchontoglires*))) is 0.013. The very low support at the deep branches may be caused by the lack of information about deep relationships of placental mammals in the molecular dataset. It may also be caused by an unreliable placement of outgroup opossum. This unsolved relationship reflects the controversies over the relationship of four major groups of placental mammals. In contrast, the Bayesian concatenation tree predominantly favors the hypothesis (*Afrotheria*, (*Xenarthra*, (*Laurasiatheria*, *Euarchontoglires*))) with a support of 0.93 [57]. The MP-EST consensus tree contains a highly improbable group of *Human *and *Strepirrhines *to the exclusion of *Tarsius*. Because the bootstrap support for this group is just 0.42, adding more data may be able to more accurately resolve the relationship among *Human*, *Strepirrhines*, and *Tarsius*.

## Conclusions

Our maximum pseudo-likelihood method, MP-EST, can consistently estimate the topology and branch lengths (in coalescent units) of the species tree including those in the anomaly zone. Although the pseudo-likelihood is derived from coalescent theory, and assumes no gene flow or horizontal gene transfer (HGT), we have shown that the MP-EST method is robust to a small number of HGT events. Unlike HGT, in which only one or a few genes might be affected, gene flow between species necessarily affects all genes in the genome, and hence potentially all trees in a data set. Thus gene flow will likely have a bigger impact on species tree estimation than will HGT. However, this situation is no different from traditional phylogenetic analysis, in which HGT and gene flow are both complicating factors [70].

MP-EST allows missing sequences for some genes. It can be used to infer species phylogenies for phylogenomic data in which it is quite common to have a substantial fraction of missing sequences. However, MP-EST may poorly perform in the presence of missing sequences in some genes. The relationship between the performance of MP-EST and the amount of missing sequences is complicated and needs further studies. The pseudo-likelihood is a function of the triplet frequencies summarized across gene trees. Since the summarized frequencies are calculated prior to the algorithm, increasing the number of genes does not increase the computational time of the algorithm for maximizing the pseudo-likelihood (Table 1). On the other hand, the computational time for calculating the likelihood function is O(N^3^) where *N *is the number of species, because the pseudo-likelihood involves $\left(\begin{array}{l} N\\ 3 \end{array}\right)$ terms. Thus increasing the number of species will certainly increase the time for finding the maximum of the pseudo-likelihood function. We tested the execution time and memory consumption for running MP-EST on a linux machine (Dual Quad Core Xeon 2.66, 32GB RAM). The execution time (CPU time) for finding the MP-EST tree of 160 species is about 51 hours (Table 2). Meanwhile, it requires at least 1.4GB memory (Table 2). The MP-EST method can quickly obtain the MPE of species trees for datasets of moderate size (≤ 80 in Table 2). For example, using a Dell PowerEdge M6000 with dual Xeon E5410 2.3 Ghz quad core processors and 32 GB RAM, it took about 40 minutes to calculate the MP-EST tree (using one CPU-core) for the reduced mammal dataset which contains 54 species and 20 genes. However, there is tremendous increase in running time for MP-EST compared to RT and STAR when more taxa are used (Table 2) although the difference in performance among the three methods is small (Figure 4).

**Table 1:** Execution times for running MP-EST as the number of genes increases.

number of genes	CPU time (seconds)		
	
	MP-EST	STAR	RT
20	22	< 1	< 1

40	23	< 1	< 1

60	25	< 1	< 1

80	20	1	< 1

Gene trees were generated from a 20-taxon species tree and used as data to reconstruct species trees using MP-EST, STAR, and RT. The analyses were conducted on a linux compute machine (Dual Quad Core Xeon 2.66, 32GB RAM).

**Table 2:** Execution times and memory consumption for running MP-EST as the number of species increases.

number of species	CPU time (seconds)			Memory (MB)		
	
	MP-EST	STAR	RT	MP-EST	STAR	RT
20	22	< 1	< 1	1421	196	8601

80	7178	3	< 1	1423	200	8610

160	185538	9	1	1436	212	8615

Twenty gene trees were generated from 20-taxon, 80-taxon, and 160-taxon species trees and used as data to reconstruct species trees using MP-EST, STAR, and RT. The analyses were conducted on a linux machine (Dual Quad Core Xeon 2.66, 32GB RAM).

Since MP-EST can estimate both the topologies and branch lengths of species trees, gene trees simulated from the MP-EST tree can be used to approximate the distribution of gene trees expected from the multispecies coalescent model. By comparing the lineage patterns in the estimated gene trees with those in the distribution of gene trees expected from the coalescent model, we can identify the lineages in the estimated gene trees that significantly deviate from the lineage patterns expected from the multispecies coalescent model. For example, if HGT occurs in the dataset, the distances among the lineages from distant species in the estimated gene trees should be significantly smaller than those expected from the multispecies coalescence model which assumes no HGT.

With regards to branch lengths, MP-EST is unable to estimate the lengths of external branches in the species tree because only one allele is sampled from each species. In addition, the internal branch length of a triple in the species tree is not estimable when all gene triples support the same topology. These internal branches are indicated with length of "99". Users should be cautious about the interpretation of length "99". It is not the actual length of the branch. The value *"*99*" *suggests that the corresponding branch length is not estimable due to the lack of topological variation among gene triples. In the cases where all genes support a single tree, MP-EST will fail to estimate any branch length.

Strategies for sampling genes are important for all species tree reconstruction methods. Biased representation of genes on the genome may introduce systematic error in species tree estimation. For example, if we would split mitochondrial genomes of placental mammals in single genes and use only these genes to estimate the species tree, MP-EST would find with bootstrap support 1.0 a species tree placing the flying lemur within primates, challenging this group paraphyletic. The MP-EST method assumes that given the species tree, the evolutionary histories of genes, i.e., gene trees, follow a coalescence process, but in practice this assumption may not always be satisfied. Although MP-EST is, to some extent, robust to the violation of the coalescent assumption, serious divergence from coalescent can certainly result in inaccurate MP-EST estimates of species trees.

Our algorithm is able at this stage to accept only a single allele per species. In addition, our algorithm yields species trees whose branch lengths are in coalescent units, rather than substitutions per site as most with most phylogenetic trees. However, such trees are still of practical use in phylogenetics. The topology of phylogenies is usually of primary interest and we have shown that the topology is consistently estimated by MP-EST. Even when we recover species trees with branch lengths in coalescent units, under certain assumptions we can obtain reasonable estimates of species divergence times in generations or years. Working in units of mutations, for example, if we assume that ancestral population sizes (θ) of lineages in our estimated species tree are similar to those of extant species (as estimated, for example, from multilocus genetic data), we can easily convert coalescent units in a species tree to branch lengths in units of substitutions per site (τ = μ). Or, when faced with variation in *θ *among extant species, one could reconstruct ancestral population sizes using any number of algorithms for phylogenetic comparative methods. Despite the fact that branch lengths are estimated in coalescent units, our algorithm is able to accommodate branch length variation in gene trees and can yield non-ultrametric species trees. In contrast, most species tree methods either ignore branch lengths in gene and/or species trees [8] or estimated ultrametric species trees [71]. Although it would be highly desirable to estimate branch lengths of species trees directly in units of substitutions per site as in traditional phylogenies and some species tree algorithms (STEM [33] and BEST [9]), such an estimation procedure would require properly modelling the mutation rate variation within and among genes. Our efforts are currently directed toward this end.

## Availability and Requirements

**Project name: **A maximum psudo-likelihood approach for estimating species trees under the coalescent model (MP-EST)

**Project home page**: http://code.google.com/p/mp-est/

**Operating system**: platform independent.

**Programming language**: C

**Other requirements**: No

**Licence**: GNU GPL.

## Authors' contributions

LL and LY developed the method and conducted the analyses. SVE obtained funding. LL, LY, and SVE drafted the manuscript. All authors read and approve the final manuscript.

## Supplementary Material

Additional file 1 Figure S1: The MP-EST tree with branch lengths for the mammal data set. Branches with length "99" (inestimable) are indicated with *. Click here for file

Additional file 2 Figure S2: The consensus MP-EST tree for the original mammal data set including the four marsupial outgroups (*opossum*, *diprotodontian*, *monitor del monte*, *shrew opossum*). Click here for file

Additional file 3 **Figure S3: The consensus gene trees for the reduced mammal data set.** We constructed a consensus tree for each of the 20 genes in the reduced mammal data set. The numbers on the branches of the consensus trees are bootstrap values based on 100 replicates. Gene trees were rooted by *opossum*. Click here for file
