# Supplementary figures and images for: A maximum pseudo-likelihood approach for estimating species trees under the coalescent model
Source: BMC Evol Biol. 2010 Oct 11;10:302. doi: 10.1186/1471-2148-10-302 (PMC2976751; doi:10.1186/1471-2148-10-302)

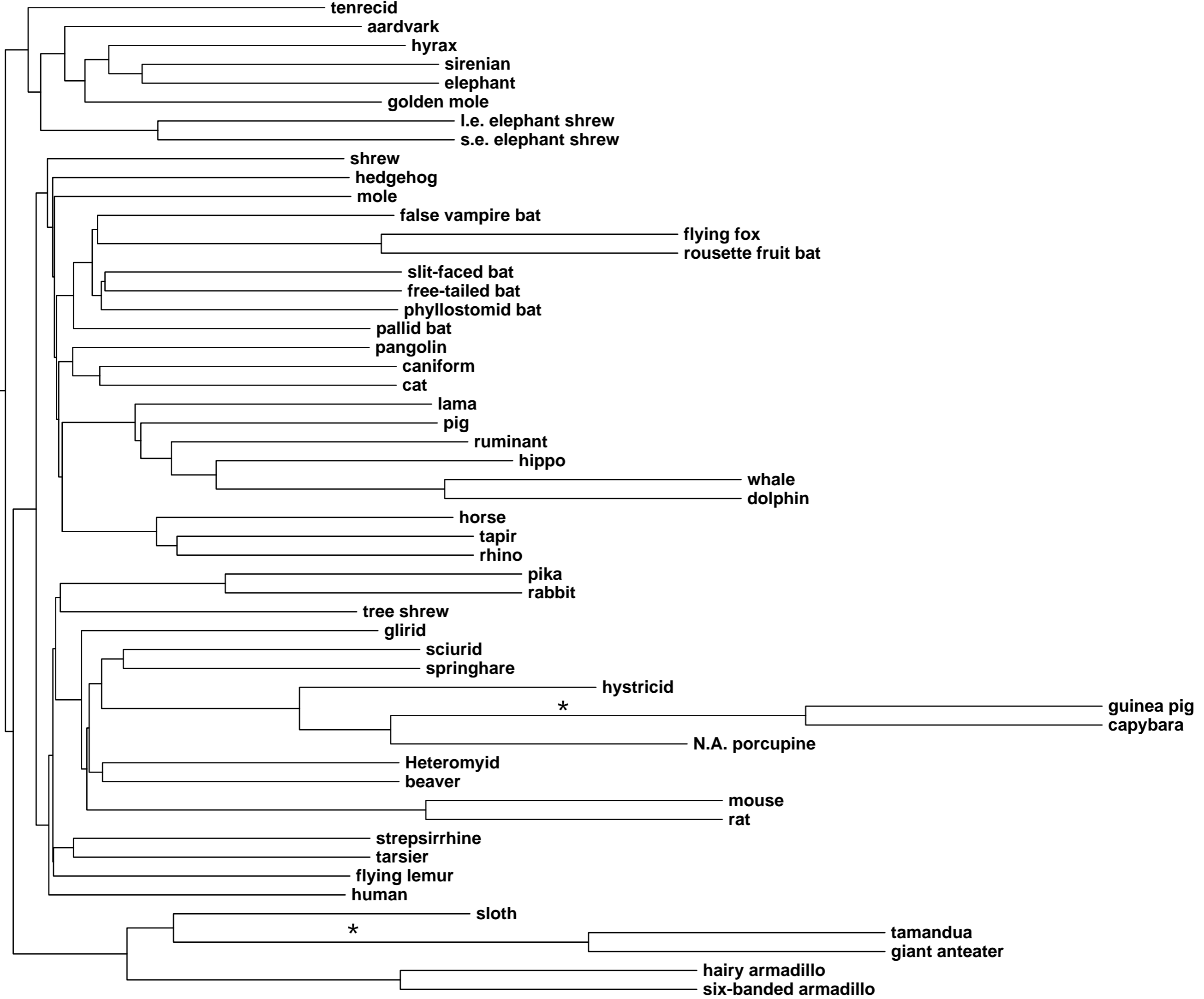

Supplement: Additional file 1 — Figure S1: The MP-EST tree with branch lengths for the mammal data set. Branches with length "99" (inestimable) are indicated with *. [file 1471-2148-10-302-S1.PDF]

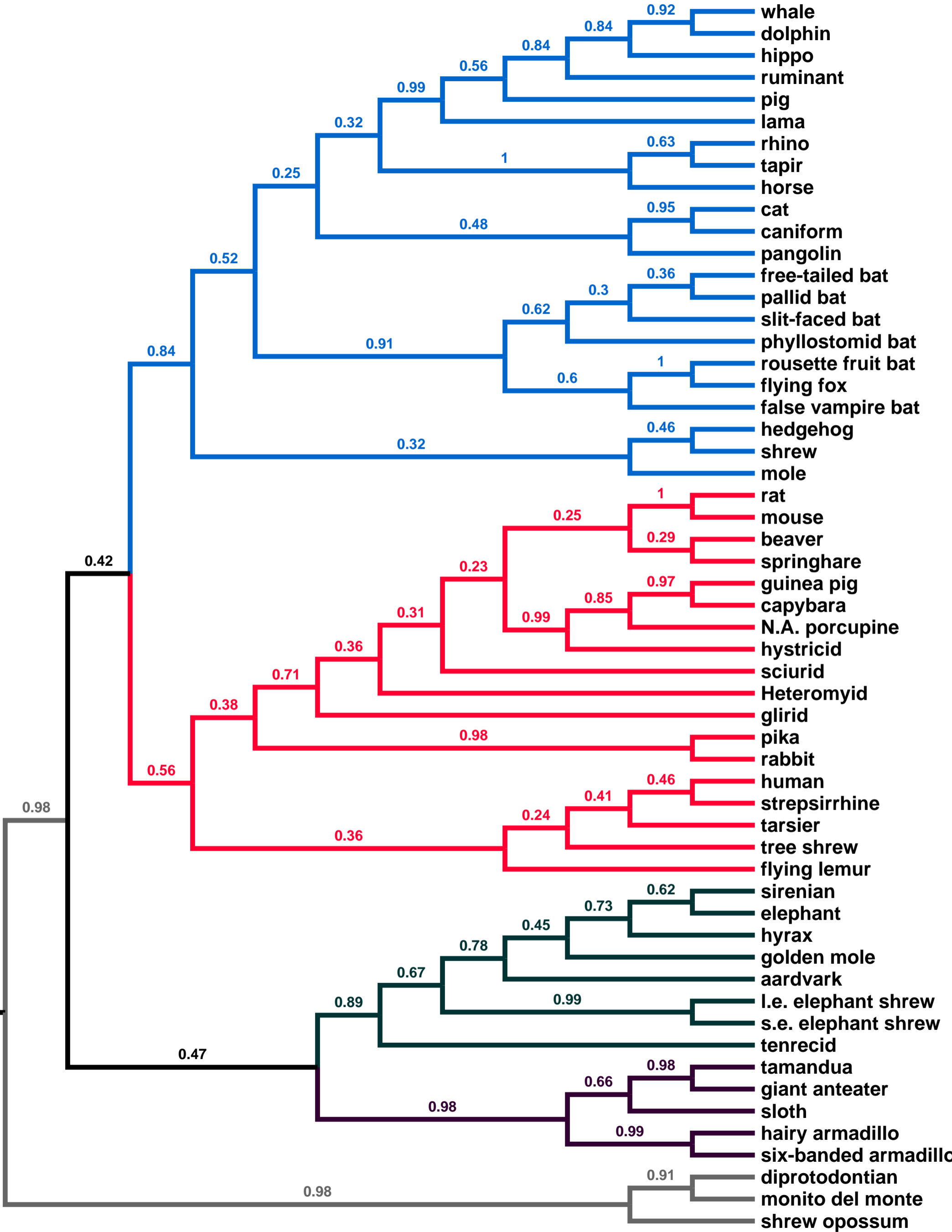

Supplement: Additional file 2 — Figure S2: The consensus MP-EST tree for the original mammal data set including the four marsupial outgroups (opossum, diprotodontian, monitor del monte, shrew opossum). [file 1471-2148-10-302-S2.PDF]
