# Supplementary material for: A maximum pseudo-likelihood approach for estimating species trees under the coalescent model
Source: BMC Evol Biol. 2010 Oct 11;10:302. doi: 10.1186/1471-2148-10-302 (PMC2976751; doi:10.1186/1471-2148-10-302)
Supplement: Additional file 3 — Figure S3: The consensus gene trees for the reduced mammal data set. We constructed a consensus tree for each of the 20 genes in the reduced mammal data set. The numbers on the branches of the consensus trees are bootstrap values based on 100 replicates. Gene trees were rooted by opossum. [file 1471-2148-10-302-S3.PDF]

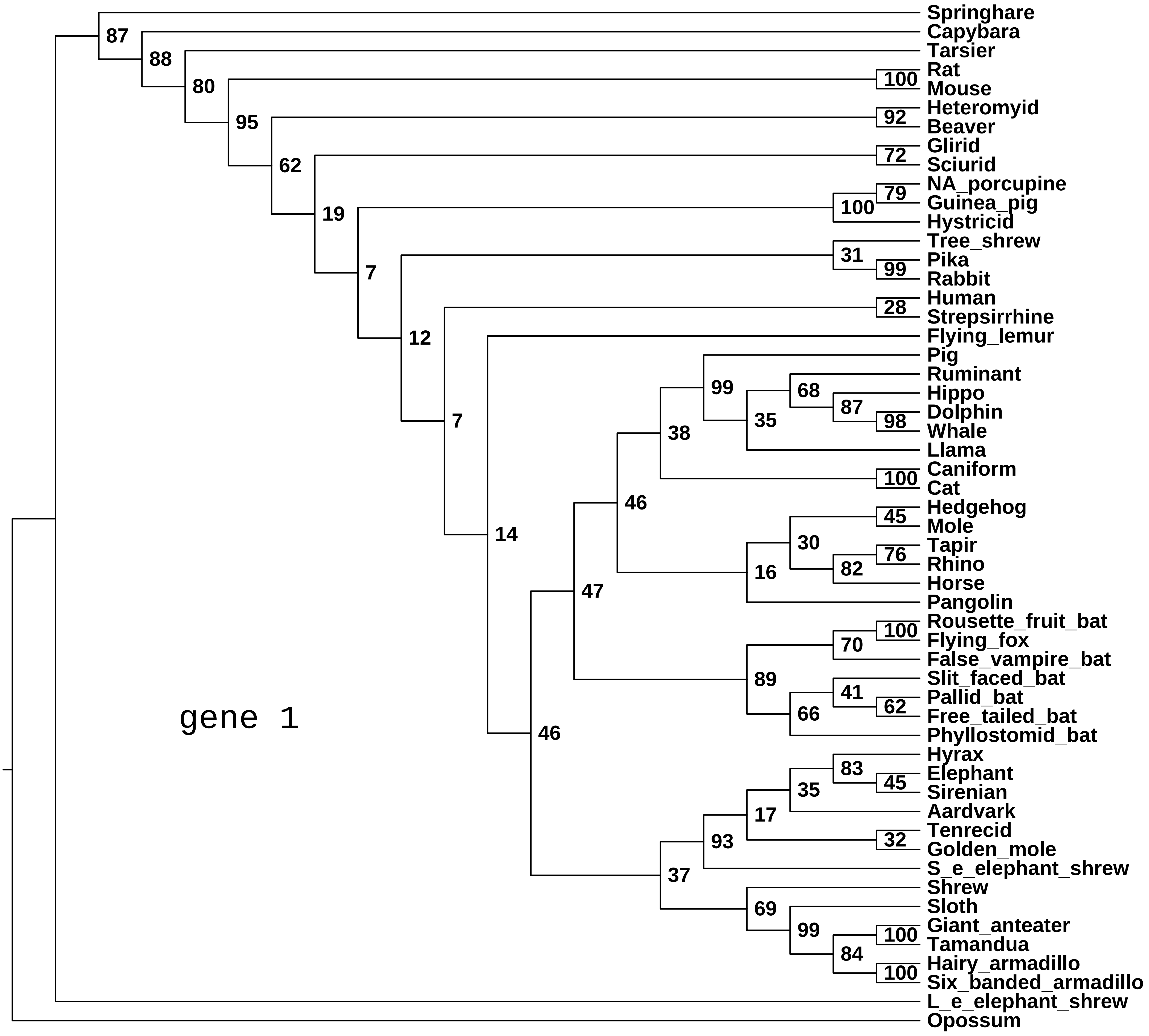
Springhare
Capybara
Tarsier
Rat
Mouse
Heteromyid
Beaver
Glirid
Sciurid
NA_porcupine
Guinea_pig
Hystricid
Tree_shrew
Pika
Rabbit
Human
Strepsirrhine
Flying_lemur
Pig
Ruminant
Hippo
Dolphin
Whale
Llama
Caniform
Cat
Hedgehog
Mole
Tapir
Rhino
Horse
Pangolin
Rousette_fruit_bat
Flying_fox
False_vampire_bat
Slit_faced_bat
Pallid_bat
Free_tailed_bat
Phyllostomid_bat
Hyrax
Elephant
Sirenian
Aardvark
Tenrecid
Golden_mole
S_e_elephant_shrew
Shrew
Sloth
Giant_anteater
Tamandua
Hairy_armadillo
Six_banded_armadillo
L_e_elephant_shrew
Opossum
gene 1

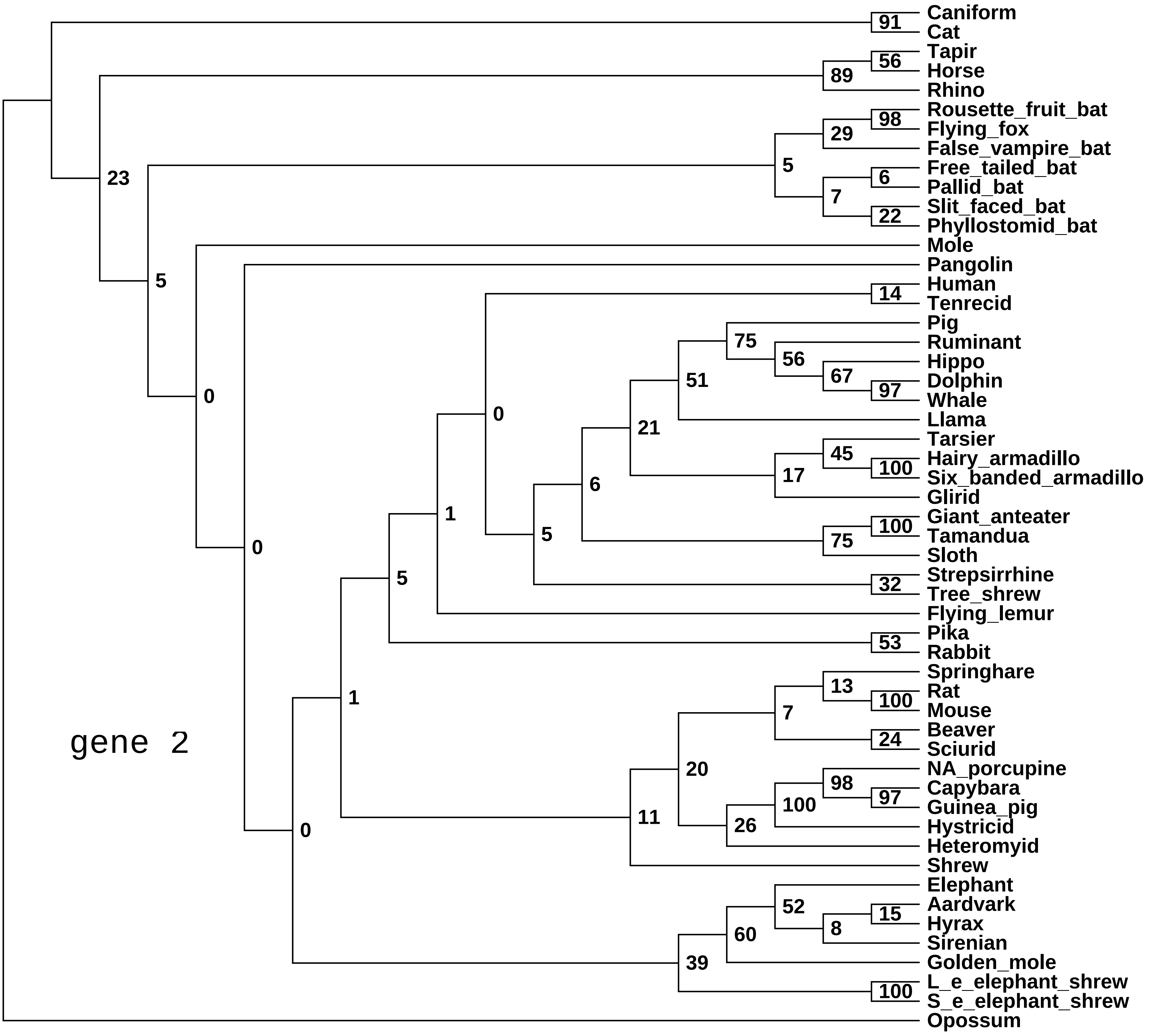

Caniform
Cat
Tapir
Horse
Rhino
Rousette_fruit_bat
Flying_fox
False_vampire_bat
Free_tailed_bat
Pallid_bat
Slit_faced_bat
Phyllostomid_bat
Mole
Pangolin
Human
Tenrecid
Pig
Ruminant
Hippo
Dolphin
Whale
Llama
Tarsier
Hairy_armadillo
Six_banded_armadillo
Glirid
Giant_anteater
Tamandua
Sloth
Strepsirrhine
Tree_shrew
Flying_lemur
Pika
Rabbit
Springhare
Rat
Mouse
Beaver
Sciurid
NA_porcupine
Capybara
Guinea_pig
Hystricid
Heteromyid
Shrew
Elephant
Aardvark
Hyrax
Sirenian
Golden_mole
L_e_elephant_shrew
S_e_elephant_shrew
Opossum
91
56
89
23
98
29
5
6
7
22
5
0
14
75
56
67
97
51
0
21
45
100
17
6
100
75
5
1
32
5
53
1
13
100
7
24
20
98
97
100
26
11
0
52
15
8
60
39
100
0
gene 2

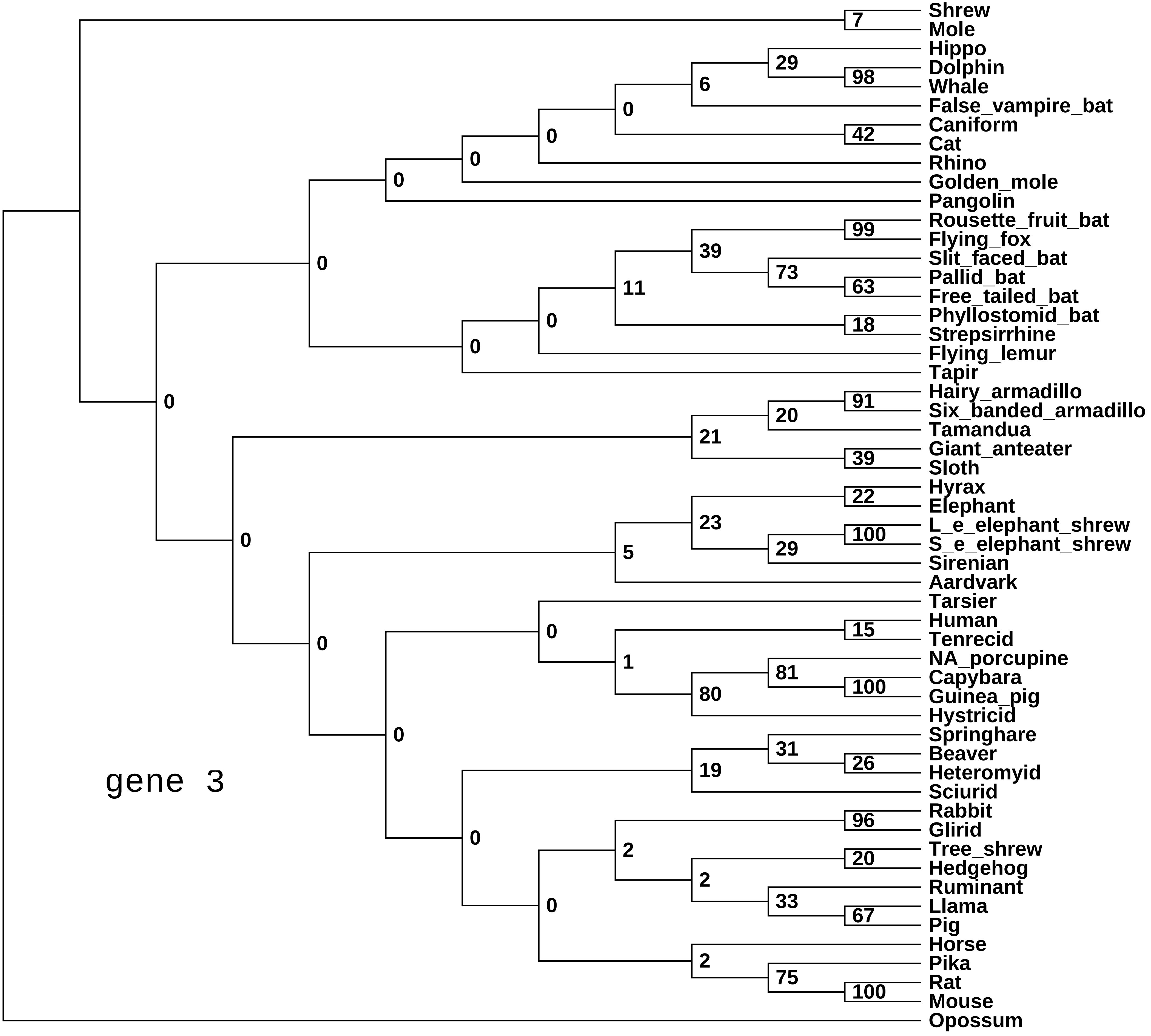

gene 3
Shrew
Mole
Hippo
Dolphin
Whale
False_vampire_bat
Caniform
Cat
Rhino
Golden_mole
Pangolin
Rousette_fruit_bat
Flying_fox
Slit_faced_bat
Pallid_bat
Free_tailed_bat
Phyllostomid_bat
Strepsirrhine
Flying_lemur
Tapir
Hairy_armadillo
Six_banded_armadillo
Tamandua
Giant_anteater
Sloth
Hyrax
Elephant
L_e_elephant_shrew
S_e_elephant_shrew
Sirenian
Aardvark
Tarsier
Human
Tenrecid
NA_porcupine
Capybara
Guinea_pig
Hystricid
Springhare
Beaver
Heteromyid
Sciurid
Rabbit
Glirid
Tree_shrew
Hedgehog
Ruminant
Llama
Pig
Horse
Pika
Rat
Mouse
Opossum

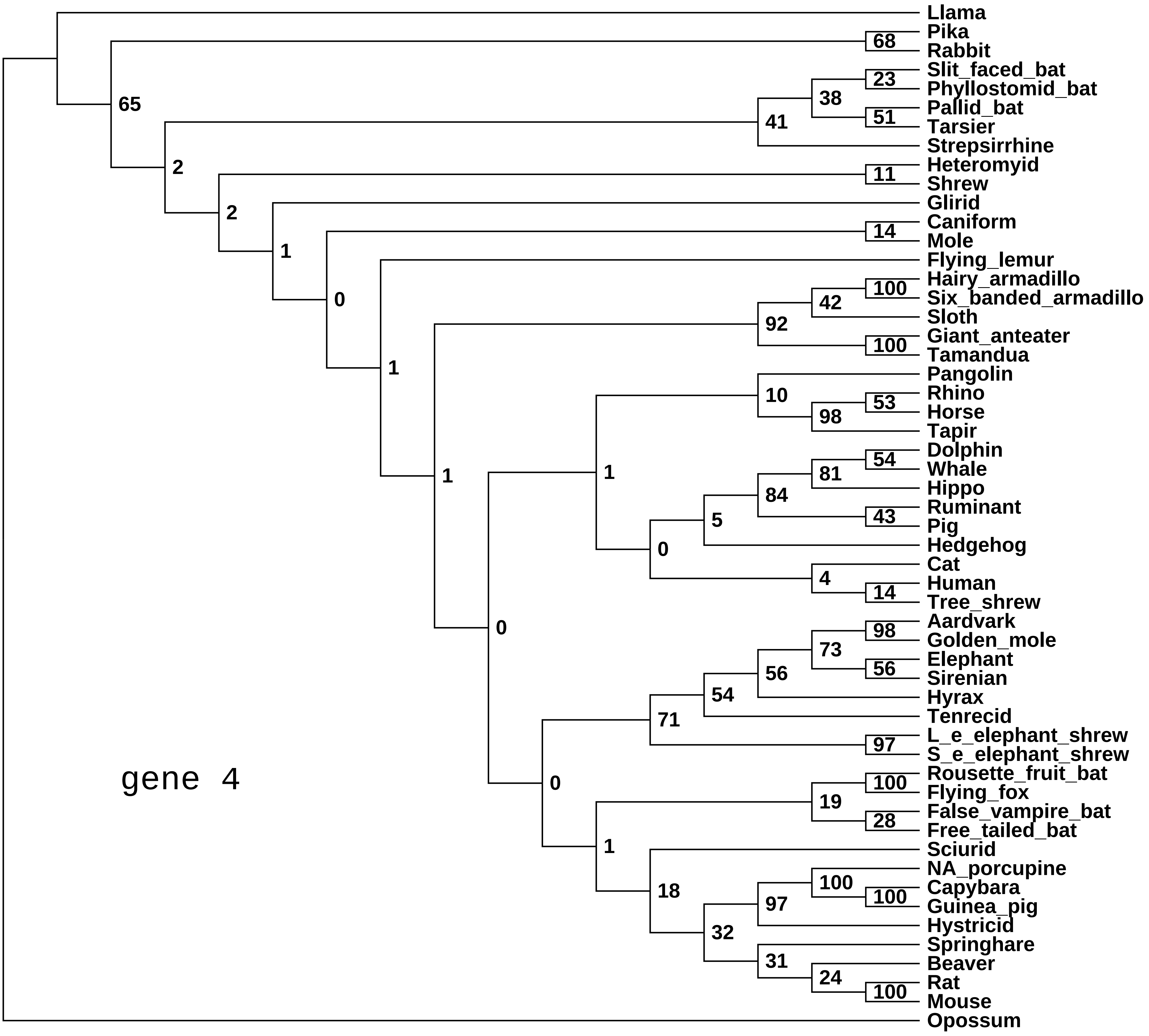

gene 4
Llama
Pika
Rabbit
Slit_faced_bat
Phyllostomid_bat
Pallid_bat
Tarsier
Strepsirrhine
Heteromyid
Shrew
Glirid
Caniform
Mole
Flying_lemur
Hairy_armadillo
Six_banded_armadillo
Sloth
Giant_anteater
Tamandua
Pangolin
Rhino
Horse
Tapir
Dolphin
Whale
Hippo
Ruminant
Pig
Hedgehog
Cat
Human
Tree_shrew
Aardvark
Golden_mole
Elephant
Sirenian
Hyrax
Tenrecid
L_e_elephant_shrew
S_e_elephant_shrew
Rousette_fruit_bat
Flying_fox
False_vampire_bat
Free_tailed_bat
Sciurid
NA_porcupine
Capybara
Guinea_pig
Hystricid
Springhare
Beaver
Rat
Mouse
Opossum

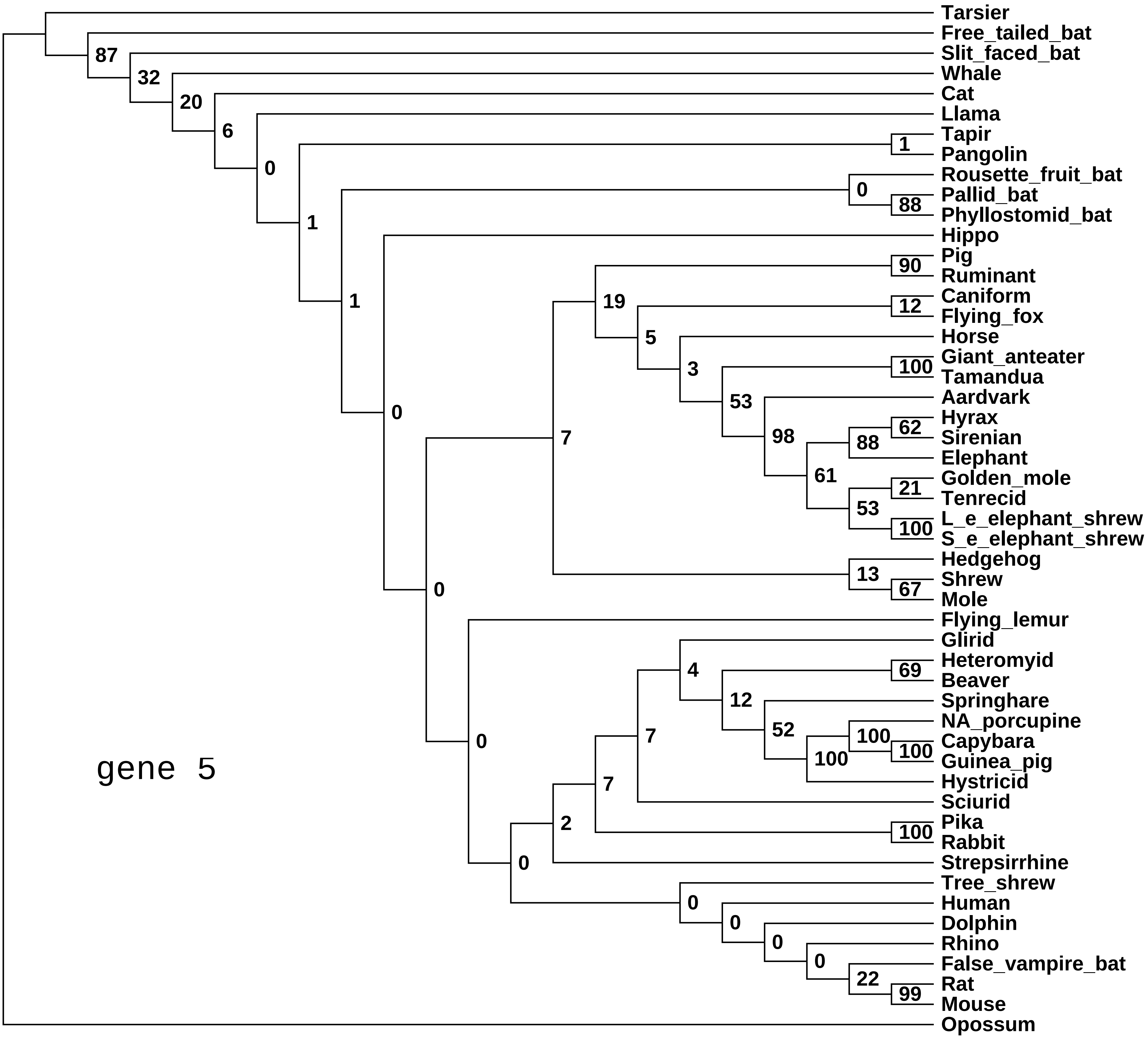
Tarsier
Free_tailed_bat
Slit_faced_bat
Whale
Cat
Llama
Tapir
Pangolin
Rousette_fruit_bat
Pallid_bat
Phyllostomid_bat
Hippo
Pig
Ruminant
Caniform
Flying_fox
Horse
Giant_anteater
Tamandua
Aardvark
Hyrax
Sirenian
Elephant
Golden_mole
Tenrecid
L_e_elephant_shrew
S_e_elephant_shrew
Hedgehog
Shrew
Mole
Flying_lemur
Glirid
Heteromyid
Beaver
Springhare
NA_porcupine
Capybara
Guinea_pig
Hystricid
Sciurid
Pika
Rabbit
Strepsirrhine
Tree_shrew
Human
Dolphin
Rhino
False_vampire_bat
Rat
Mouse
Opossum
gene 5

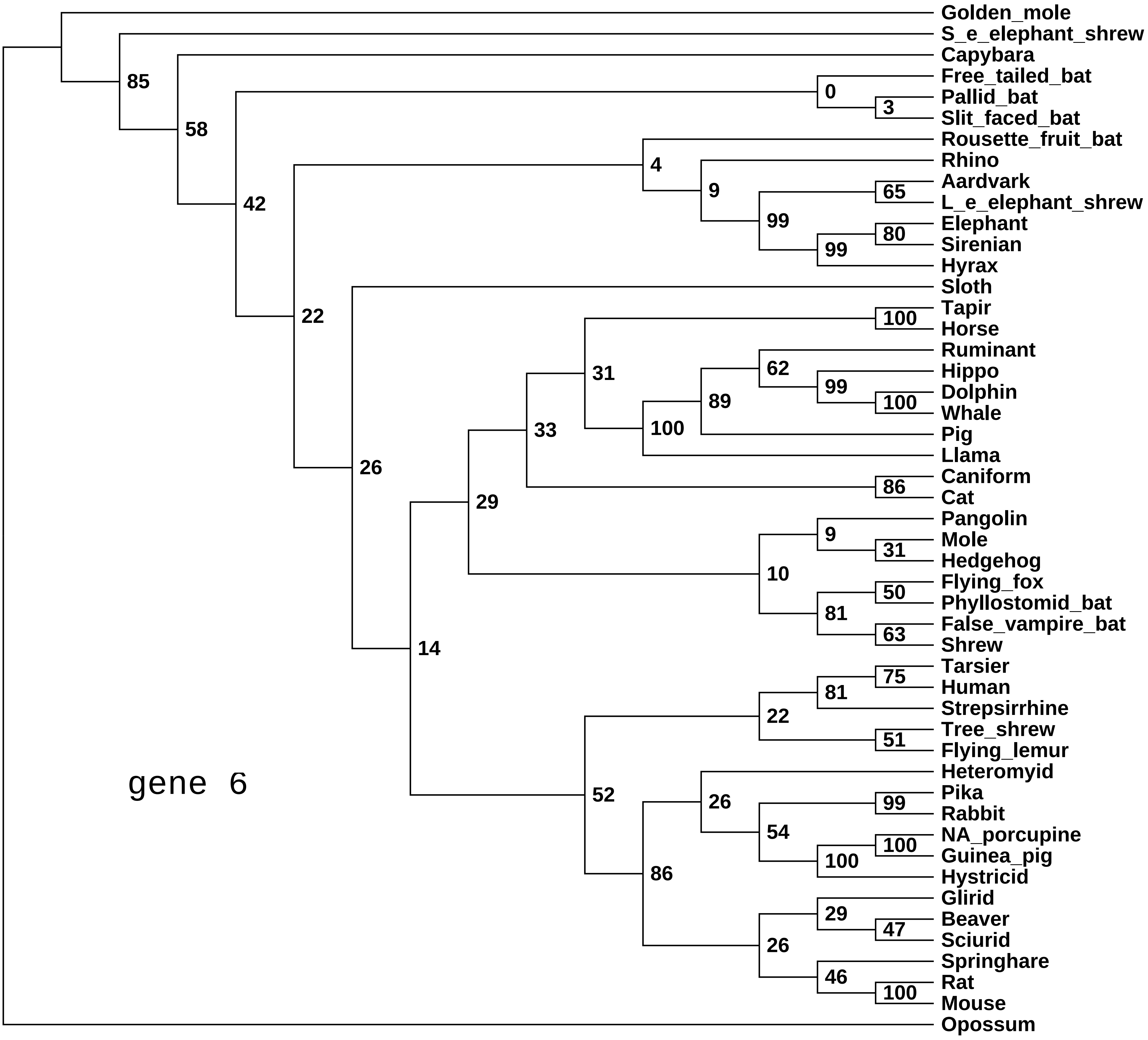
Golden_mole
S_e_elephant_shrew
Capybara
Free_tailed_bat
Pallid_bat
Slit_faced_bat
Rousette_fruit_bat
Rhino
Aardvark
L_e_elephant_shrew
Elephant
Sirenian
Hyrax
Sloth
Tapir
Horse
Ruminant
Hippo
Dolphin
Whale
Pig
Llama
Caniform
Cat
Pangolin
Mole
Hedgehog
Flying_fox
Phyllostomid_bat
False_vampire_bat
Shrew
Tarsier
Human
Strepsirrhine
Tree_shrew
Flying_lemur
Heteromyid
Pika
Rabbit
NA_porcupine
Guinea_pig
Hystricid
Glirid
Beaver
Sciurid
Springhare
Rat
Mouse
Opossum
gene 6

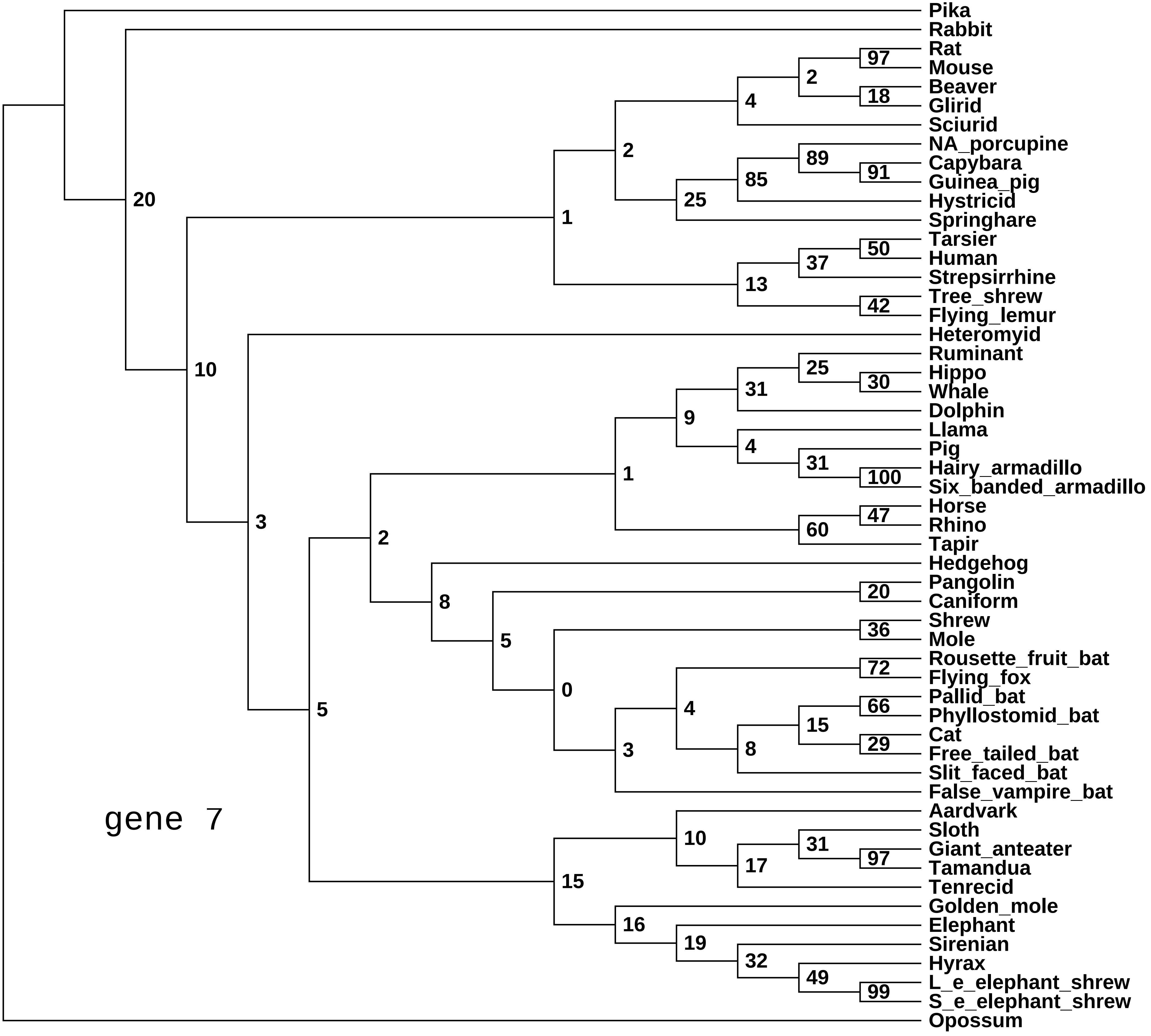
Pika
Rabbit
Rat
Mouse
Beaver
Glirid
Sciurid
NA_porcupine
Capybara
Guinea_pig
Hystricid
Springhare
Tarsier
Human
Strepsirrhine
Tree_shrew
Flying_lemur
Heteromyid
Ruminant
Hippo
Whale
Dolphin
Llama
Pig
Hairy_armadillo
Six_banded_armadillo
Horse
Rhino
Tapir
Hedgehog
Pangolin
Caniform
Shrew
Mole
Rousette_fruit_bat
Flying_fox
Pallid_bat
Phyllostomid_bat
Cat
Free_tailed_bat
Slit_faced_bat
False_vampire_bat
Aardvark
Sloth
Giant_anteater
Tamandua
Tenrecid
Golden_mole
Elephant
Sirenian
Hyrax
L_e_elephant_shrew
S_e_elephant_shrew
Opossum
gene 7

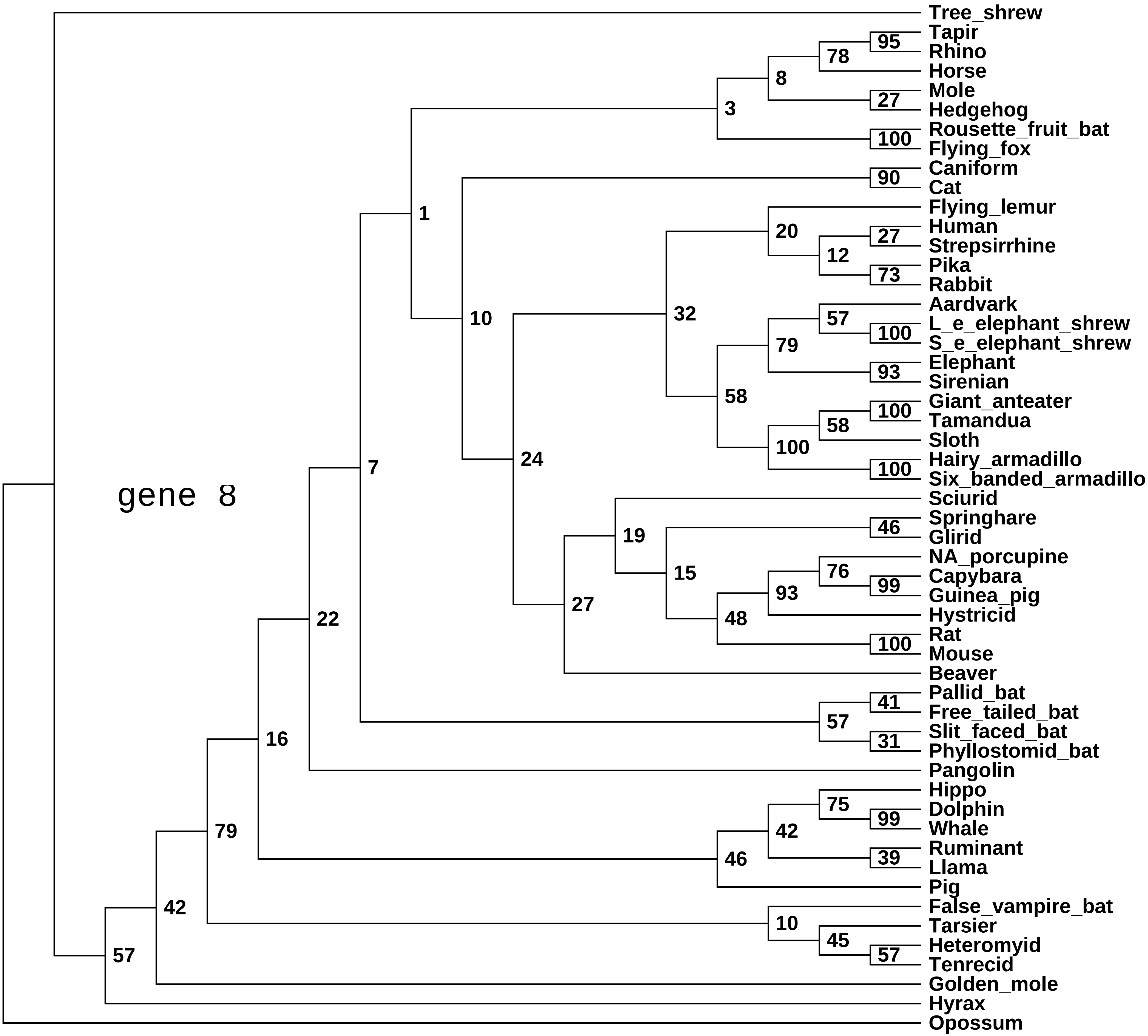
gene 8
Tree_shrew
Tapir
Rhino
Horse
Mole
Hedgehog
Rousette_fruit_bat
Flying_fox
Caniform
Cat
Flying_lemur
Human
Strepsirrhine
Pika
Rabbit
Aardvark
L_e_elephant_shrew
S_e_elephant_shrew
Elephant
Sirenian
Giant_anteater
Tamandua
Sloth
Hairy_armadillo
Six_banded_armadillo
Sciurid
Springhare
Glirid
NA_porcupine
Capybara
Guinea_pig
Hystricid
Rat
Mouse
Beaver
Pallid_bat
Free_tailed_bat
Slit_faced_bat
Phyllostomid_bat
Pangolin
Hippo
Dolphin
Whale
Ruminant
Llama
Pig
False_vampire_bat
Tarsier
Heteromyid
Tenrecid
Golden_mole
Hyrax
Opossum

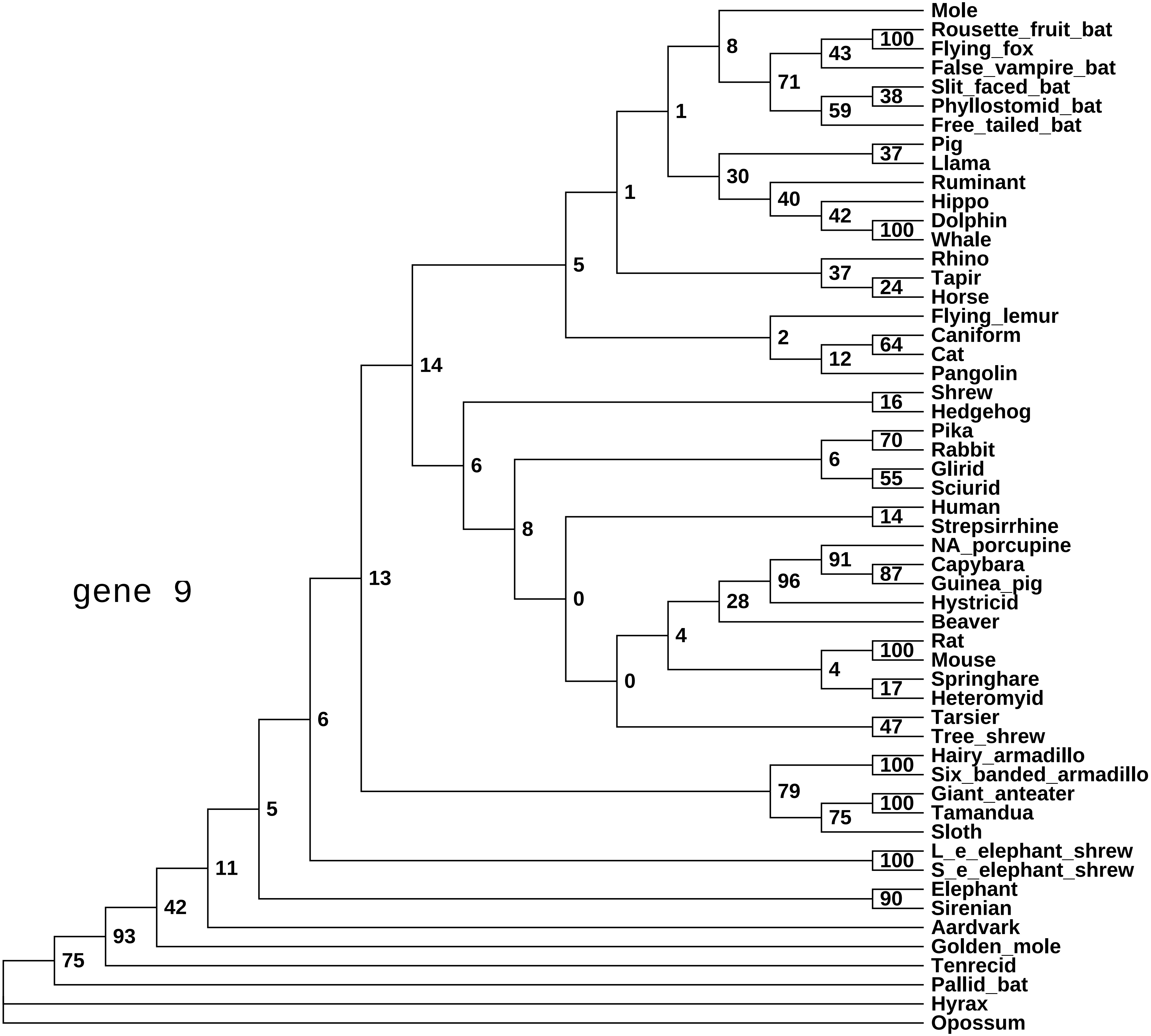
gene 9
Mole
Rousette_fruit_bat
Flying_fox
False_vampire_bat
Slit_faced_bat
Phyllostomid_bat
Free_tailed_bat
Pig
Llama
Ruminant
Hippo
Dolphin
Whale
Rhino
Tapir
Horse
Flying_lemur
Caniform
Cat
Pangolin
Shrew
Hedgehog
Pika
Rabbit
Glirid
Sciurid
Human
Strepsirrhine
NA_porcupine
Capybara
Guinea_pig
Hystricid
Beaver
Rat
Mouse
Springhare
Heteromyid
Tarsier
Tree_shrew
Hairy_armadillo
Six_banded_armadillo
Giant_anteater
Tamandua
Sloth
L_e_elephant_shrew
S_e_elephant_shrew
Elephant
Sirenian
Aardvark
Golden_mole
Tenrecid
Pallid_bat
Hyrax
Opossum

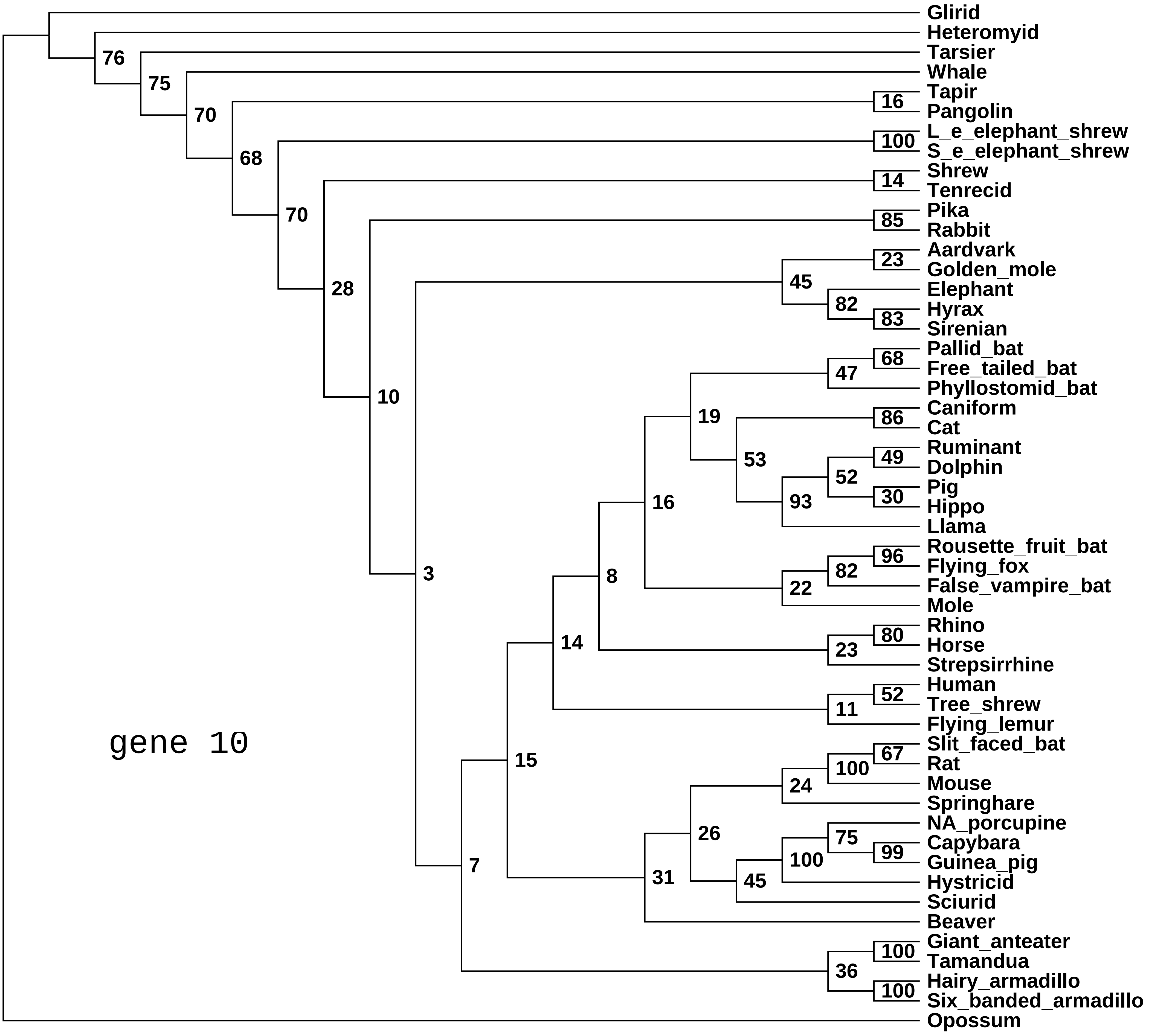
Glirid
Heteromyid
Tarsier
Whale
Tapir
Pangolin
L_e_elephant_shrew
S_e_elephant_shrew
Shrew
Tenrecid
Pika
Rabbit
Aardvark
Golden_mole
Elephant
Hyrax
Sirenian
Pallid_bat
Free_tailed_bat
Phyllostomid_bat
Caniform
Cat
Ruminant
Dolphin
Pig
Hippo
Llama
Rousette_fruit_bat
Flying_fox
False_vampire_bat
Mole
Rhino
Horse
Strepsirrhine
Human
Tree_shrew
Flying_lemur
Slit_faced_bat
Rat
Mouse
Springhare
NA_porcupine
Capybara
Guinea_pig
Hystricid
Sciurid
Beaver
Giant_anteater
Tamandua
Hairy_armadillo
Six_banded_armadillo
Opossum
76
75
70
68
70
28
10
3
7
15
14
8
16
19
53
93
52
47
45
82
22
23
11
31
26
24
45
100
36
16
100
14
85
23
83
68
86
49
30
96
80
52
67
75
99
100
100
gene 10

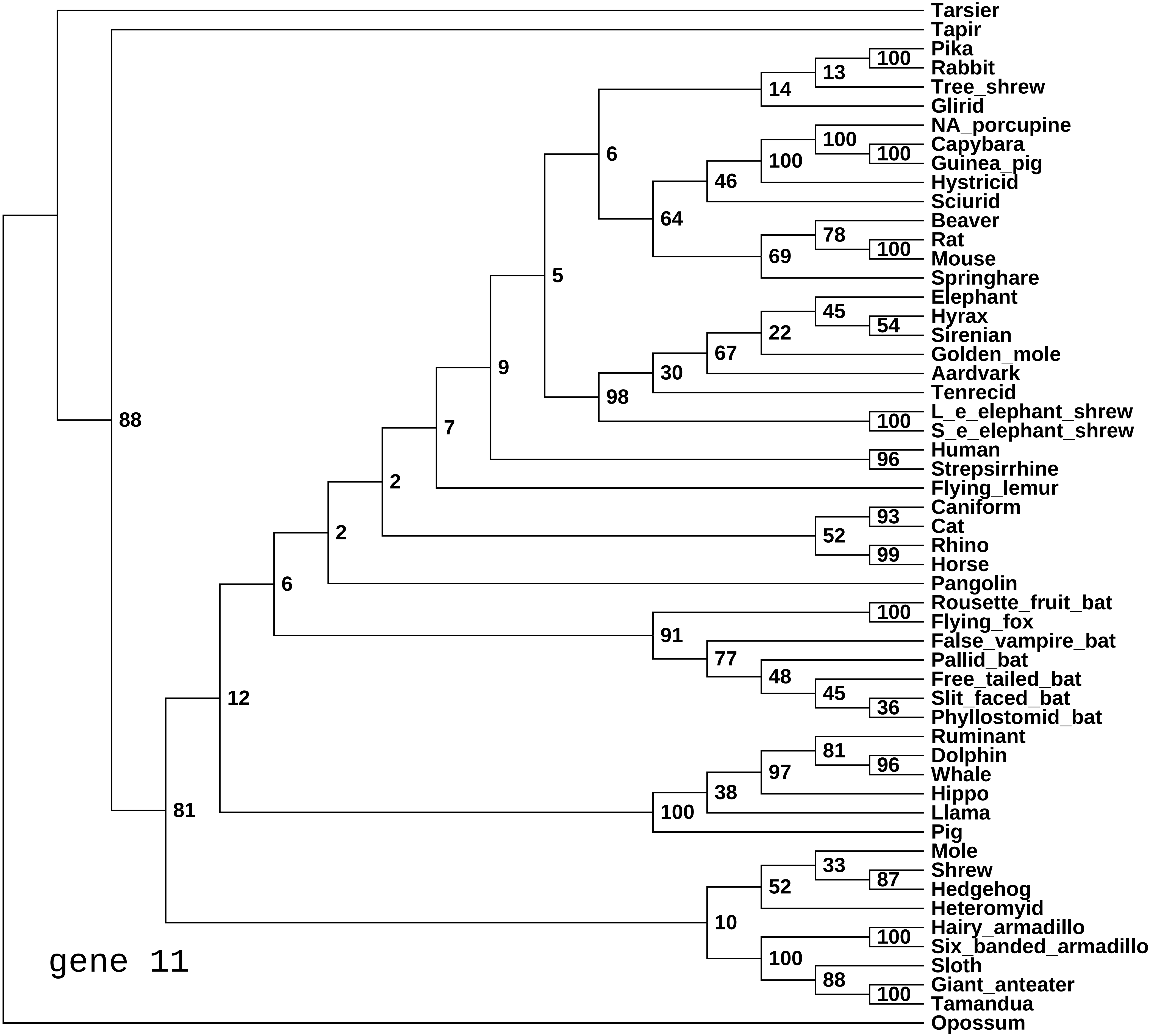
Tarsier
Tapir
Pika
Rabbit
Tree_shrew
Glirid
NA_porcupine
Capybara
Guinea_pig
Hystricid
Sciurid
Beaver
Rat
Mouse
Springhare
Elephant
Hyrax
Sirenian
Golden_mole
Aardvark
Tenrecid
L_e_elephant_shrew
S_e_elephant_shrew
Human
Strepsirrhine
Flying_lemur
Caniform
Cat
Rhino
Horse
Pangolin
Rousette_fruit_bat
Flying_fox
False_vampire_bat
Pallid_bat
Free_tailed_bat
Slit_faced_bat
Phyllostomid_bat
Ruminant
Dolphin
Whale
Hippo
Llama
Pig
Mole
Shrew
Hedgehog
Heteromyid
Hairy_armadillo
Six_banded_armadillo
Sloth
Giant_anteater
Tamandua
Opossum
100
13
14
100
100
100
6
46
64
78
100
69
5
45
54
22
67
30
98
100
9
96
7
2
93
52
99
2
6
100
91
77
48
45
36
12
88
81
96
97
38
100
81
33
87
52
10
100
100
88
100

gene 11

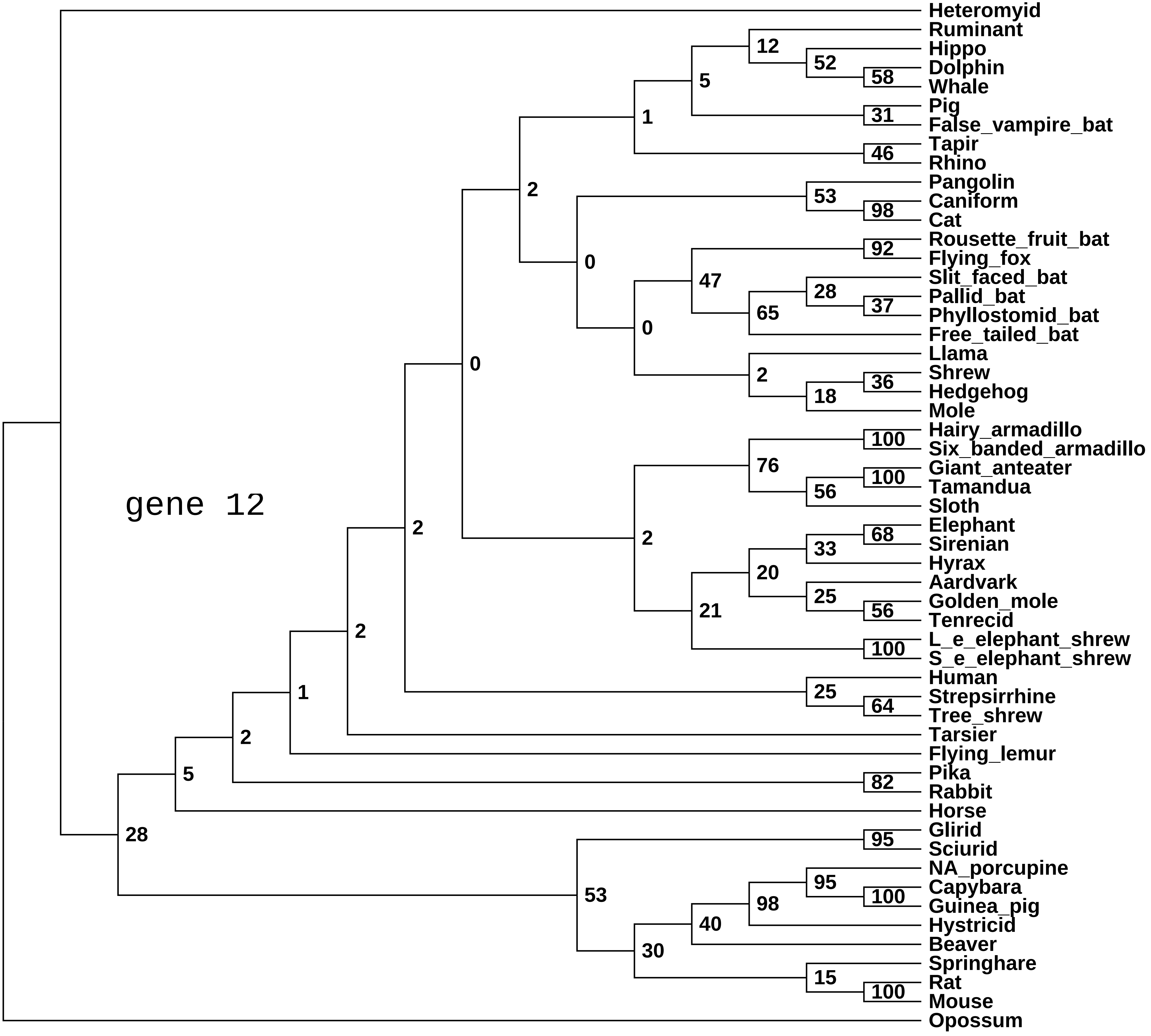

gene 12
Heteromyid
Ruminant
Hippo
Dolphin
Whale
Pig
False_vampire_bat
Tapir
Rhino
Pangolin
Caniform
Cat
Rousette_fruit_bat
Flying_fox
Slit_faced_bat
Pallid_bat
Phyllostomid_bat
Free_tailed_bat
Llama
Shrew
Hedgehog
Mole
Hairy_armadillo
Six_banded_armadillo
Giant_anteater
Tamandua
Sloth
Elephant
Sirenian
Hyrax
Aardvark
Golden_mole
Tenrecid
L_e_elephant_shrew
S_e_elephant_shrew
Human
Strepsirrhine
Tree_shrew
Tarsier
Flying_lemur
Pika
Rabbit
Horse
Glirid
Sciurid
NA_porcupine
Capybara
Guinea_pig
Hystricid
Beaver
Springhare
Rat
Mouse
Opossum

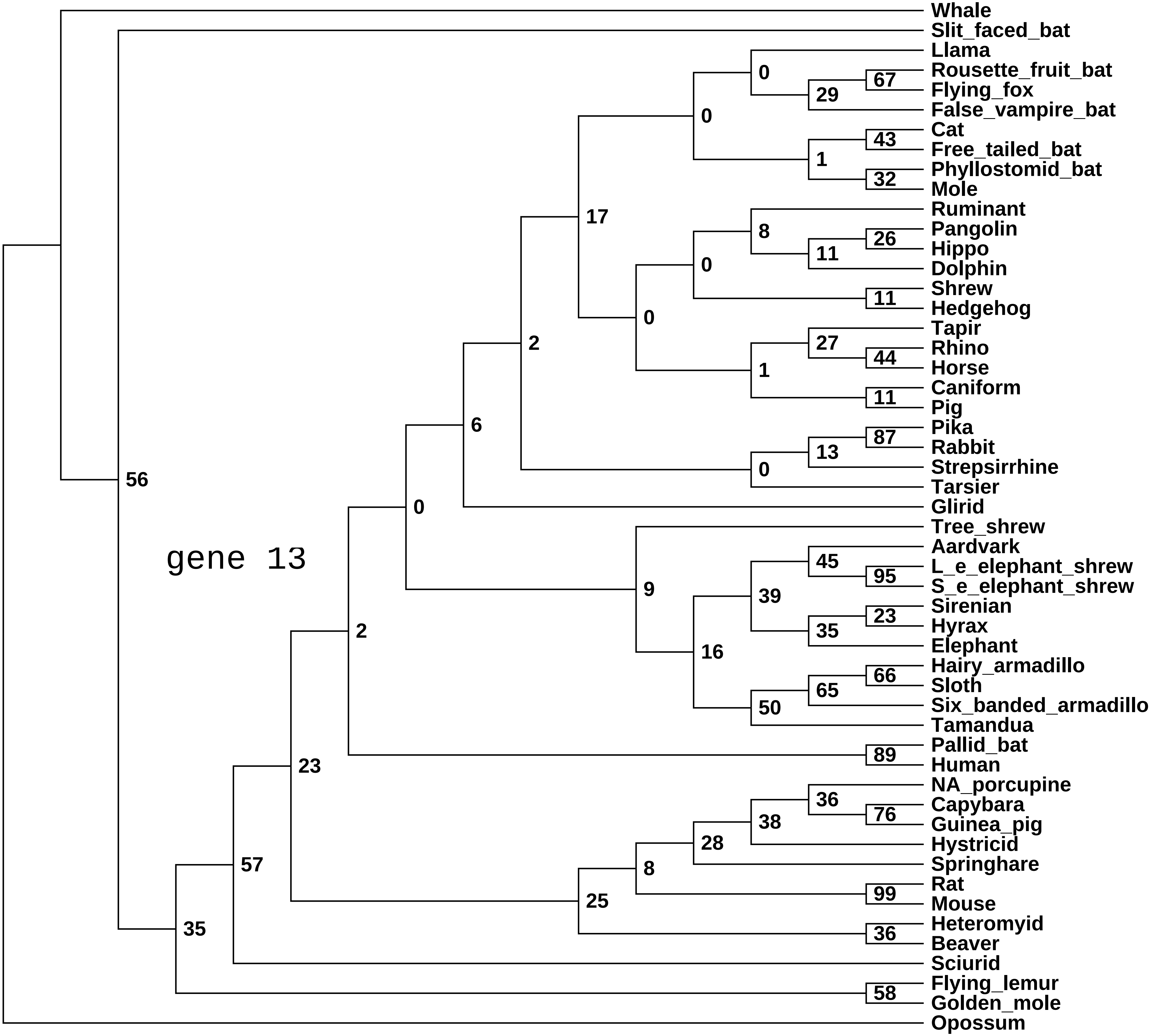
gene 13
Whale
Slit_faced_bat
Llama
Rousette_fruit_bat
Flying_fox
False_vampire_bat
Cat
Free_tailed_bat
Phyllostomid_bat
Mole
Ruminant
Pangolin
Hippo
Dolphin
Shrew
Hedgehog
Tapir
Rhino
Horse
Caniform
Pig
Pika
Rabbit
Strepsirrhine
Tarsier
Glirid
Tree_shrew
Aardvark
L_e_elephant_shrew
S_e_elephant_shrew
Sirenian
Hyrax
Elephant
Hairy_armadillo
Sloth
Six_banded_armadillo
Tamandua
Pallid_bat
Human
NA_porcupine
Capybara
Guinea_pig
Hystricid
Springhare
Rat
Mouse
Heteromyid
Beaver
Sciurid
Flying_lemur
Golden_mole
Opossum

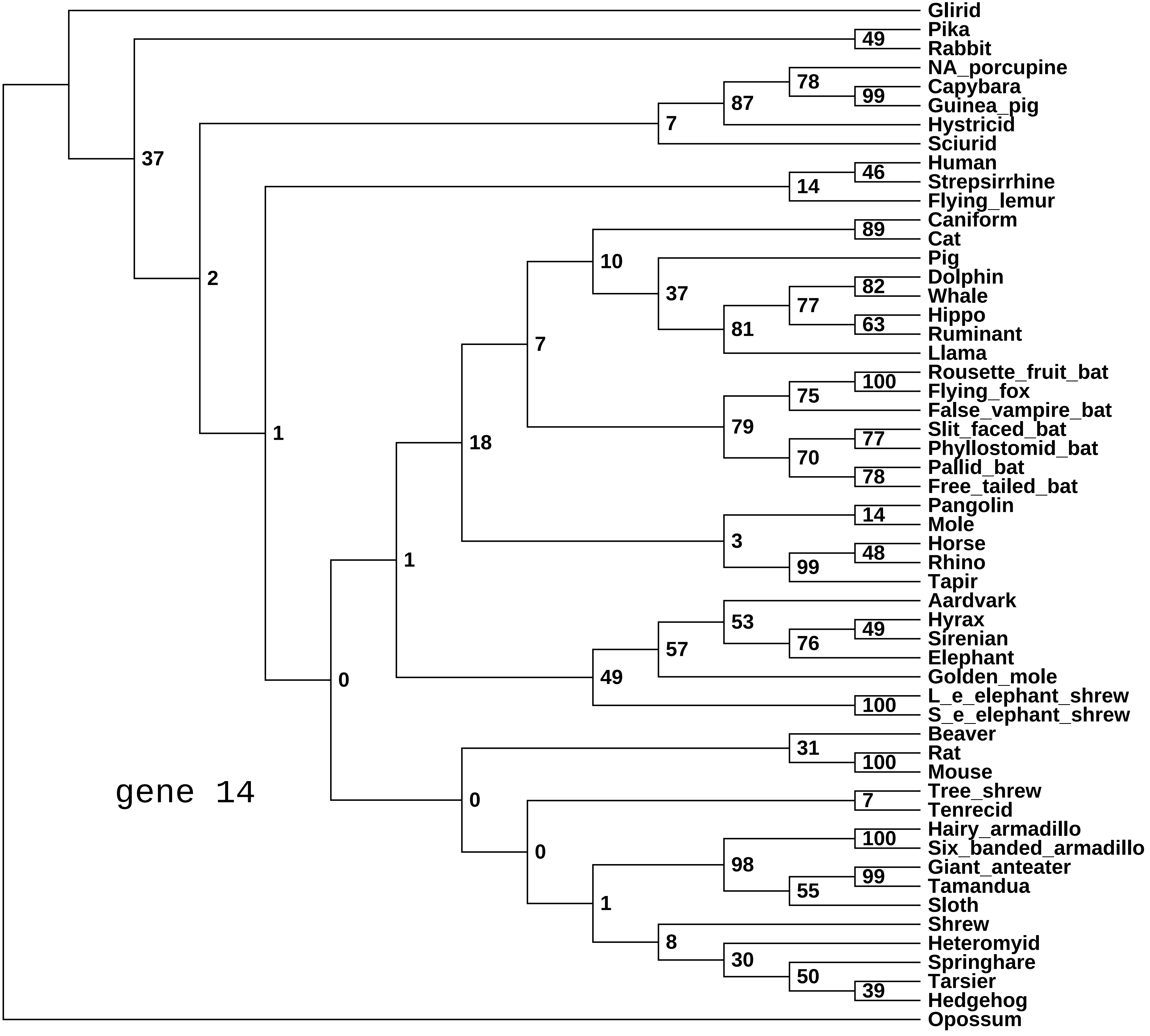
Glirid
Pika
Rabbit
NA_porcupine
Capybara
Guinea_pig
Hystricid
Sciurid
Human
Strepsirrhine
Flying_lemur
Caniform
Cat
Pig
Dolphin
Whale
Hippo
Ruminant
Llama
Rousette_fruit_bat
Flying_fox
False_vampire_bat
Slit_faced_bat
Phyllostomid_bat
Pallid_bat
Free_tailed_bat
Pangolin
Mole
Horse
Rhino
Tapir
Aardvark
Hyrax
Sirenian
Elephant
Golden_mole
L_e_elephant_shrew
S_e_elephant_shrew
Beaver
Rat
Mouse
Tree_shrew
Tenrecid
Hairy_armadillo
Six_banded_armadillo
Giant_anteater
Tamandua
Sloth
Shrew
Heteromyid
Springhare
Tarsier
Hedgehog
Opossum
49
78
99
87
7
37
46
14
89
10
82
77
63
81
37
7
100
75
79
77
70
78
18
14
3
48
99
1
53
49
76
57
49
100
2
1
0
31
100
7
0
100
98
99
55
1
8
30
50
39
0
gene 14

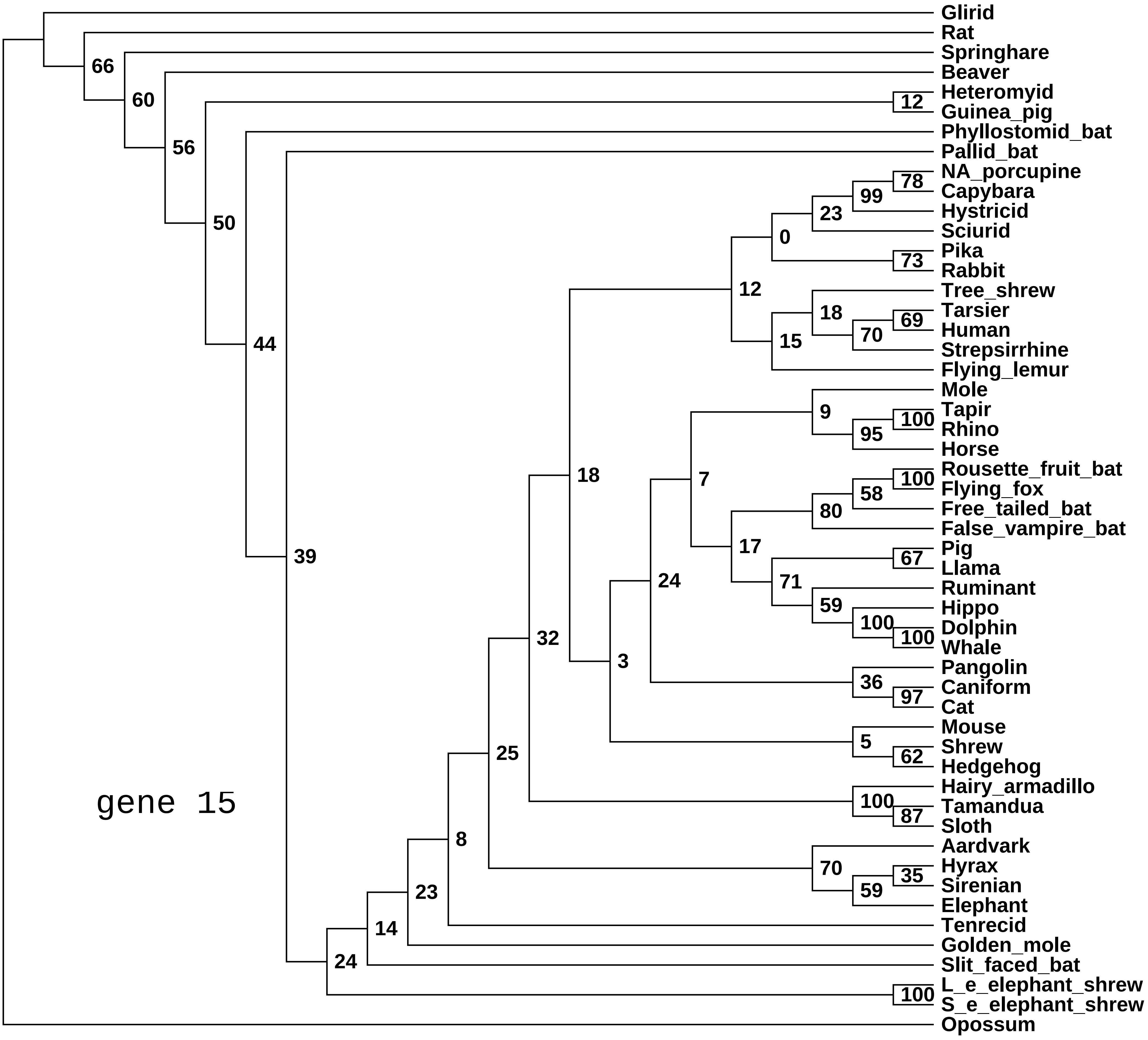
Glirid
Rat
Springhare
Beaver
Heteromyid
Guinea_pig
Phyllostomid_bat
Pallid_bat
NA_porcupine
Capybara
Hystricid
Sciurid
Pika
Rabbit
Tree_shrew
Tarsier
Human
Strepsirrhine
Flying_lemur
Mole
Tapir
Rhino
Horse
Rousette_fruit_bat
Flying_fox
Free_tailed_bat
False_vampire_bat
Pig
Llama
Ruminant
Hippo
Dolphin
Whale
Pangolin
Caniform
Cat
Mouse
Shrew
Hedgehog
Hairy_armadillo
Tamandua
Sloth
Aardvark
Hyrax
Sirenian
Elephant
Tenrecid
Golden_mole
Slit_faced_bat
L_e_elephant_shrew
S_e_elephant_shrew
Opossum
66
60
56
50
44
39
12
78
99
23
0
73
12
18
69
70
15
9
100
95
18
7
100
58
80
17
67
71
59
100
100
24
32
3
36
97
5
62
25
100
87
8
70
35
59
23
14
24
100
gene 15

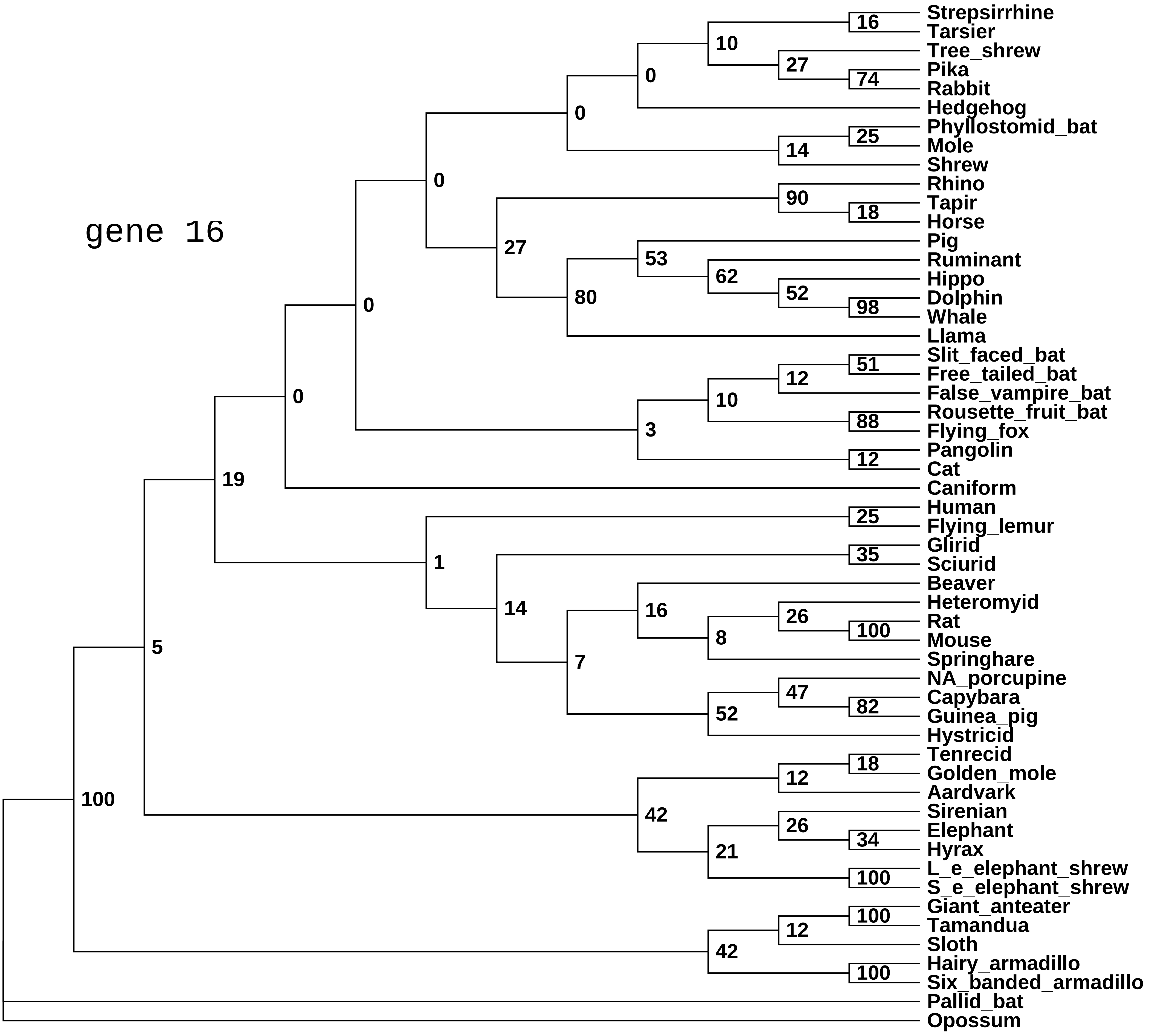
gene 16
Strepsirrhine
Tarsier
Tree_shrew
Pika
Rabbit
Hedgehog
Phyllostomid_bat
Mole
Shrew
Rhino
Tapir
Horse
Pig
Ruminant
Hippo
Dolphin
Whale
Llama
Slit_faced_bat
Free_tailed_bat
False_vampire_bat
Rousette_fruit_bat
Flying_fox
Pangolin
Cat
Caniform
Human
Flying_lemur
Glirid
Sciurid
Beaver
Heteromyid
Rat
Mouse
Springhare
NA_porcupine
Capybara
Guinea_pig
Hystricid
Tenrecid
Golden_mole
Aardvark
Sirenian
Elephant
Hyrax
L_e_elephant_shrew
S_e_elephant_shrew
Giant_anteater
Tamandua
Sloth
Hairy_armadillo
Six_banded_armadillo
Pallid_bat
Opossum

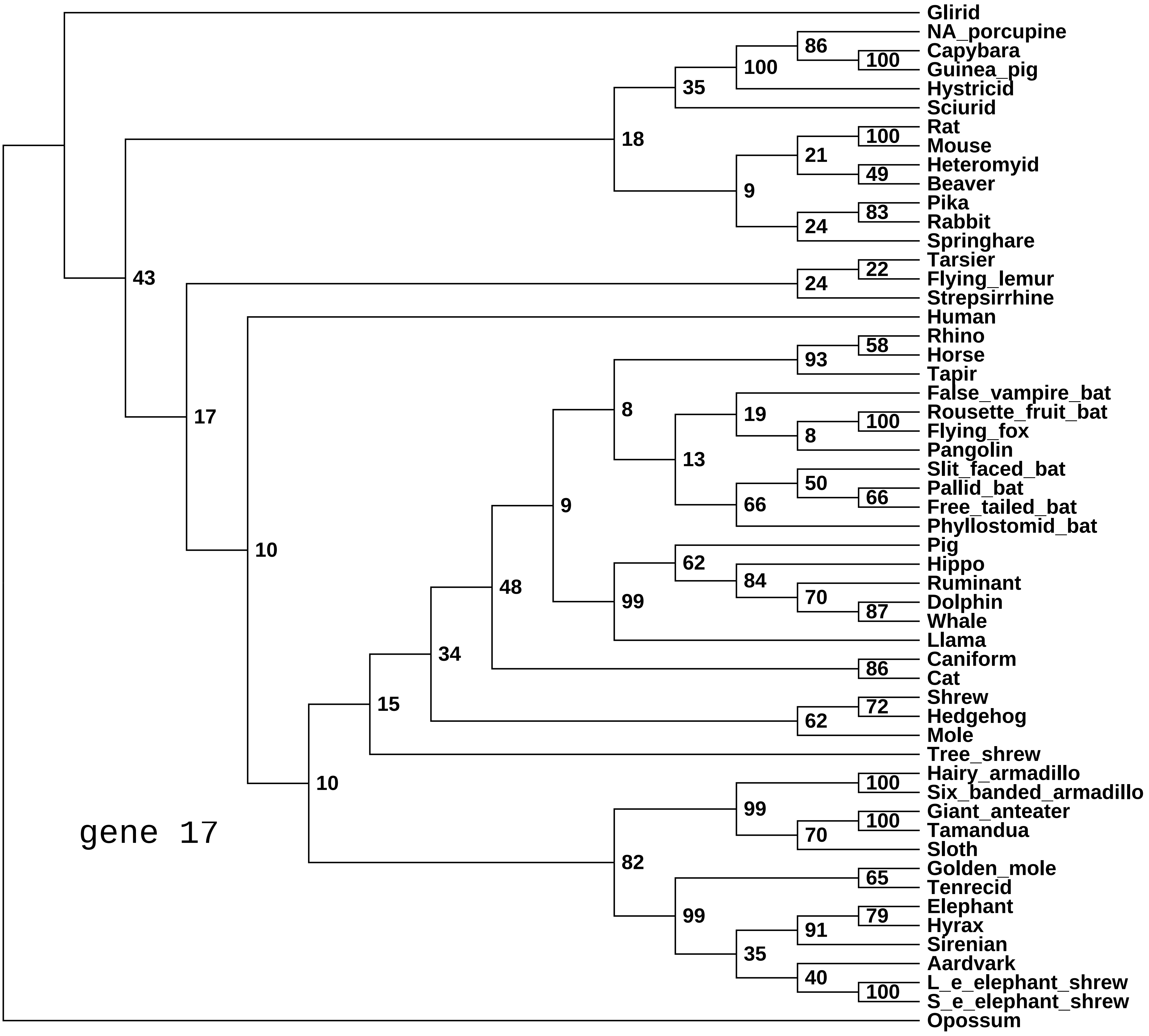
Glirid
NA_porcupine
Capybara
Guinea_pig
Hystricid
Sciurid
Rat
Mouse
Heteromyid
Beaver
Pika
Rabbit
Springhare
Tarsier
Flying_lemur
Strepsirrhine
Human
Rhino
Horse
Tapir
False_vampire_bat
Rousette_fruit_bat
Flying_fox
Pangolin
Slit_faced_bat
Pallid_bat
Free_tailed_bat
Phyllostomid_bat
Pig
Hippo
Ruminant
Dolphin
Whale
Llama
Caniform
Cat
Shrew
Hedgehog
Mole
Tree_shrew
Hairy_armadillo
Six_banded_armadillo
Giant_anteater
Tamandua
Sloth
Golden_mole
Tenrecid
Elephant
Hyrax
Sirenian
Aardvark
L_e_elephant_shrew
S_e_elephant_shrew
Opossum
gene 17

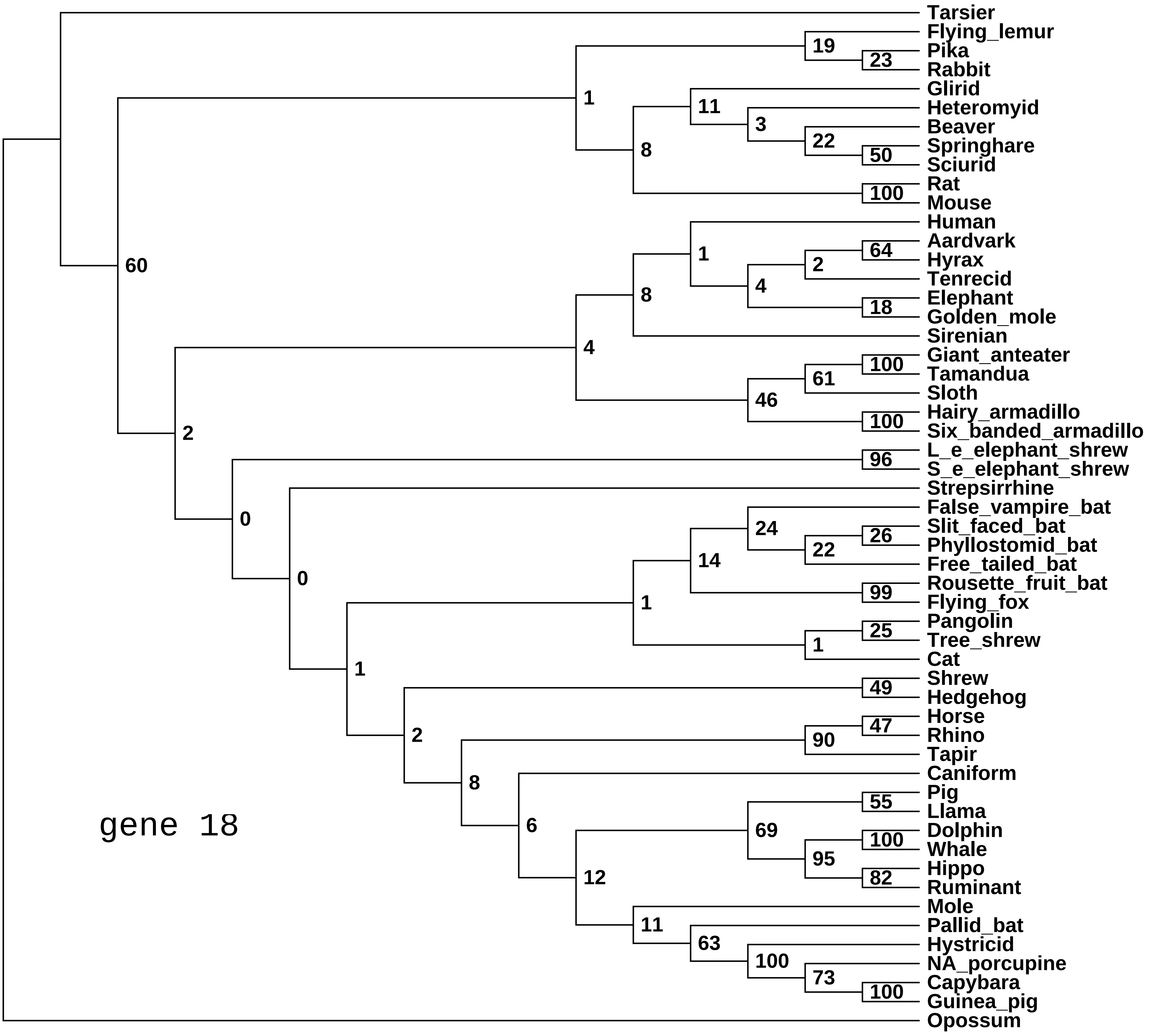
Tarsier
Flying_lemur
Pika
Rabbit
Glirid
Heteromyid
Beaver
Springhare
Sciurid
Rat
Mouse
Human
Aardvark
Hyrax
Tenrecid
Elephant
Golden_mole
Sirenian
Giant_anteater
Tamandua
Sloth
Hairy_armadillo
Six_banded_armadillo
L_e_elephant_shrew
S_e_elephant_shrew
Strepsirrhine
False_vampire_bat
Slit_faced_bat
Phyllostomid_bat
Free_tailed_bat
Rousette_fruit_bat
Flying_fox
Pangolin
Tree_shrew
Cat
Shrew
Hedgehog
Horse
Rhino
Tapir
Caniform
Pig
Llama
Dolphin
Whale
Hippo
Ruminant
Mole
Pallid_bat
Hystricid
NA_porcupine
Capybara
Guinea_pig
Opossum
19
23
1
11
3
22
50
8
100
60
1
64
2
4
18
8
4
100
61
46
100
2
96
0
24
26
22
14
99
1
25
1
0
1
49
47
90
2
8
55
69
100
95
82
6
12
11
63
100
73
100
gene 18

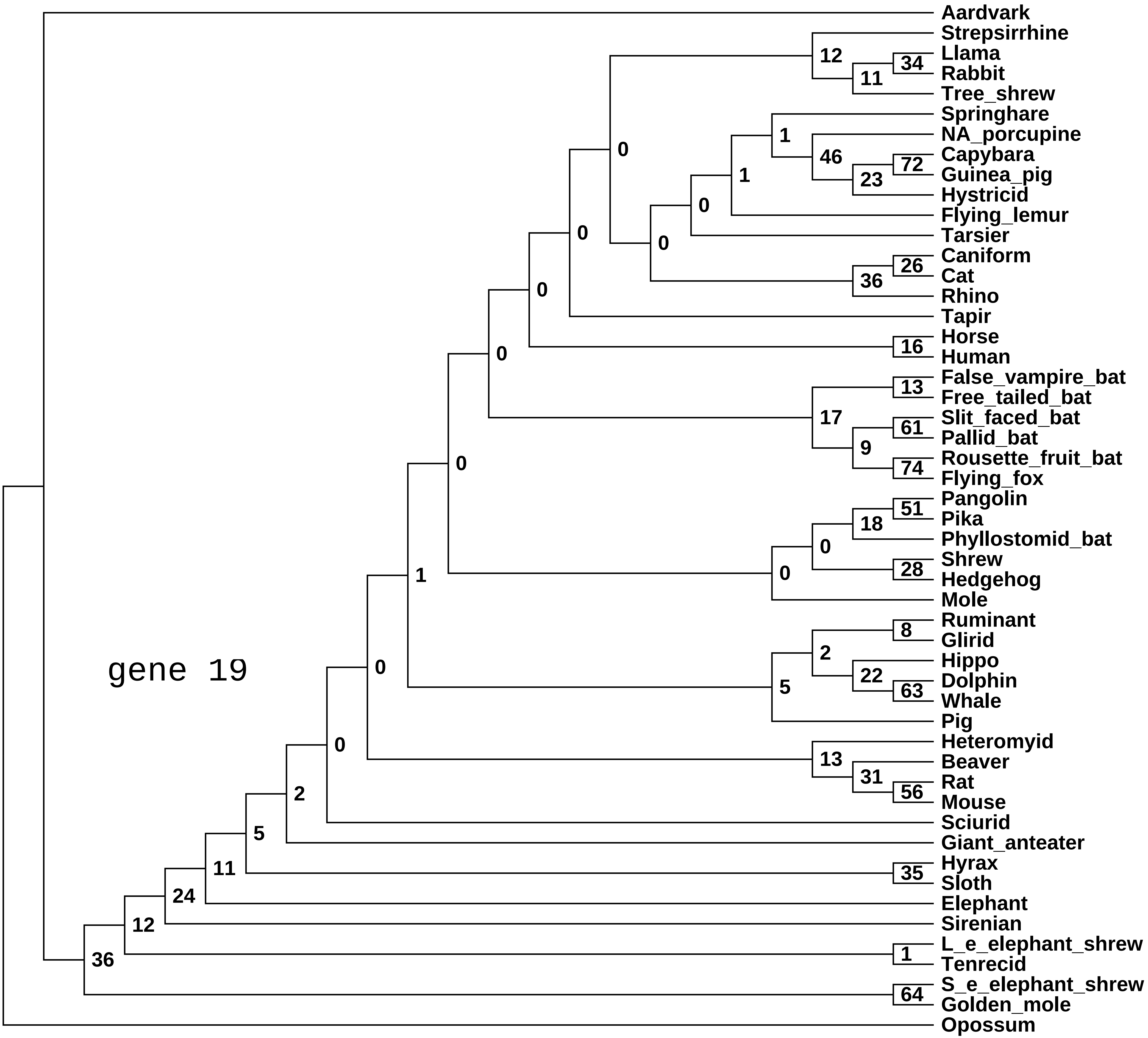
gene 19
Aardvark
Strepsirrhine
Llama
Rabbit
Tree_shrew
Springhare
NA_porcupine
Capybara
Guinea_pig
Hystricid
Flying_lemur
Tarsier
Caniform
Cat
Rhino
Tapir
Horse
Human
False_vampire_bat
Free_tailed_bat
Slit_faced_bat
Pallid_bat
Rousette_fruit_bat
Flying_fox
Pangolin
Pika
Phyllostomid_bat
Shrew
Hedgehog
Mole
Ruminant
Glirid
Hippo
Dolphin
Whale
Pig
Heteromyid
Beaver
Rat
Mouse
Sciurid
Giant_anteater
Hyrax
Sloth
Elephant
Sirenian
L_e_elephant_shrew
Tenrecid
S_e_elephant_shrew
Golden_mole
Opossum

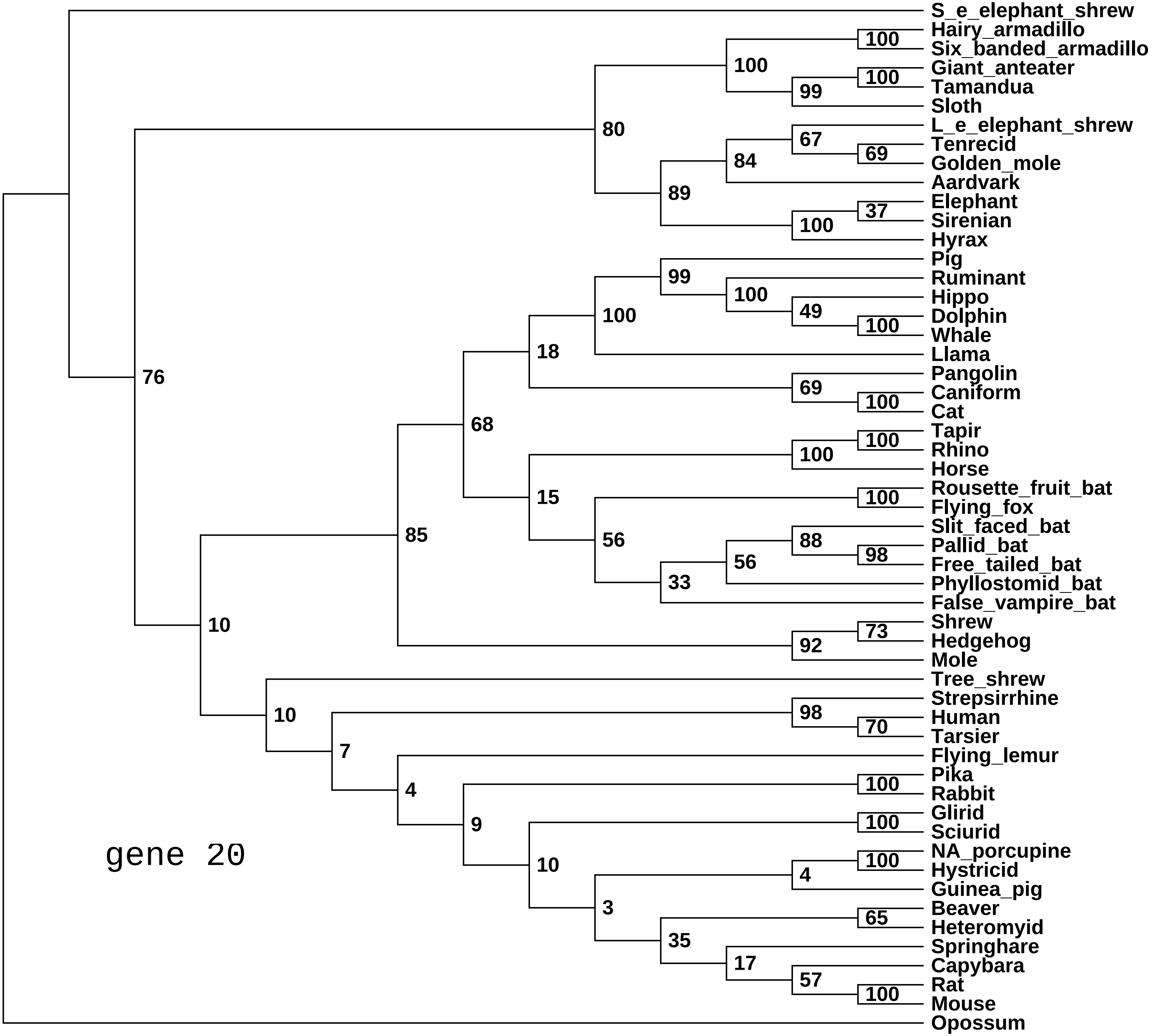
S_e_elephant_shrew
Hairy_armadillo
Six_banded_armadillo
Giant_anteater
Tamandua
Sloth
L_e_elephant_shrew
Tenrecid
Golden_mole
Aardvark
Elephant
Sirenian
Hyrax
Pig
Ruminant
Hippo
Dolphin
Whale
Llama
Pangolin
Caniform
Cat
Tapir
Rhino
Horse
Rousette_fruit_bat
Flying_fox
Slit_faced_bat
Pallid_bat
Free_tailed_bat
Phyllostomid_bat
False_vampire_bat
Shrew
Hedgehog
Mole
Tree_shrew
Strepsirrhine
Human
Tarsier
Flying_lemur
Pika
Rabbit
Glirid
Sciurid
NA_porcupine
Hystricid
Guinea_pig
Beaver
Heteromyid
Springhare
Capybara
Rat
Mouse
Opossum
100
100
99
100
80
67
69
84
89
37
100
76
99
100
49
100
100
18
69
100
68
100
100
15
100
56
88
98
56
33
85
73
92
10
10
98
70
7
4
100
9
100
10
100
4
3
65
35
17
57
100
gene 20
